# Anion Exchange Mechanism of PFAS with Cholestyramine: Implications for PFAS Removal from the Human Body

**DOI:** 10.3390/toxics14100901

**Published:** 2026-10-11

**Authors:** Ronan Zwa, Sahith Mada, Sivanesan Dakshanamurthy

**Affiliations:** 1 Department of Molecular Biology, Cell Biology, and Biochemistry, Brown University, Providence, RI 02912, USA; 2 College of Computer, Mathematical, and Natural Sciences, University of Maryland, College Park, MD 20742, USA; 3 Lombardi Comprehensive Cancer Center, Georgetown University Medical Center, Washington, DC 20007, USA; 4 Innscite AI LLC, Herndon, VA 20170, USA

**Keywords:** PFAS, PFOS, PFOA, cholestyramine, anion exchange resin, DFT, ωB97X-D3, r^2^SCAN-3c, SMD/CPCM, enterohepatic recirculation, ion pairing, backbone contacts

## Abstract

Per- and polyfluoroalkyl substances (PFAS) can bioaccumulate and may increase the risk of cancer development. Clinical studies report that cholestyramine treatment lowers serum PFAS concentrations, but the molecular energetics, interactions and underlying mechanisms of PFAS exchange with the resin remain incompletely understood. Here, we present a quantum perspective to define the mechanism of PFAS anion exchange with cholestyramine. Quantum chemistry calculations, including density functional theory (DFT), were used to evaluate chloride exchange for perfluorooctanesulfonic acid (PFOS), perfluorooctanoic acid (PFOA), perfluorohexanoic acid (PFHxA), and 6:2 fluorotelomer carboxylic acid (6:2 FTCA) at an isolated ammonium site and in an extended styrene-divinylbenzene model. Single-site ion pairing did not make exchange with chloride thermodynamically favorable. Adding the divinylbenzene backbone lowered electronic exchange energies for all four PFAS, whereas the Gibbs free-energy effect varied among compounds. Energy decomposition analysis showed that electrostatic attraction was the largest stabilizing contribution, while added backbone contacts altered dispersion, exchange correlation and Pauli contributions. For PFOA, changing the bound state continuum from water to 1-octanol produced little net change in exchange free energy. Three separately seeded 10 ns molecular dynamics trajectories of a hydrated pVBTMA_12_ oligomer showed persistent PFOA contact with changing headgroup and tail environments, although global structural convergence was not established. Together, these results support a molecular mechanism in which electrostatic ion pairing and PFAS backbone contacts jointly influence anion exchange favorability in simplified cholestyramine models. This mechanistic framework provides testable hypotheses for PFAS sequestration and identifies aromatic contact area and ammonium site geometry as design variables for future resin optimization. These findings connect quantum level interaction energetics with experimentally testable strategies for developing improved PFAS sequestrants. Computational data and code are archived in Zenodo, and a web-based graphical user interface tool is available online.

## 1. Introduction

Per- and polyfluoroalkyl substances (PFAS) enter the human body through contaminated food and water [1]. After absorption, many PFAS bind serum albumin and other transport proteins. PFAS can also undergo enterohepatic recirculation, in which compounds are secreted from the liver into the intestine via bile and are reabsorbed into the blood [2]. Repeated circulation contributes to long biological half-lives [3]. PFAS exposure has been linked to increased cancer risk, as well as poor endocrine and immune function [4,5,6,7]. PFAS removal from circulation is thus a significant clinical challenge.

Renal clearance is inefficient for many PFAS, so limiting intestinal reabsorption is one approach to PFAS removal. Cholestyramine is a quaternary ammonium anion exchange resin used to bind bile acids in the gastrointestinal tract [8]. It contains cationic exchange sites that are normally paired with chloride counterions. Since PFAS are anionic at physiological pH, cholestyramine treatment can be modeled as competition between PFAS anions and chloride near cationic sites. Clinical studies report that cholestyramine lowers serum PFAS concentrations [9,10]. These data are consistent with PFAS binding to cholestyramine in the gut. Experimental adsorption studies can measure PFAS removal capacity for activated carbon and synthetic polymers [11]. Computational models based on descriptors can also predict sorption trends [12]. However, the molecular interactions that cause cholestyramine to favor PFAS over chloride have not been fully explored. A molecular model of anion exchange can examine the local interactions involved in sequestration.

Experimental PFOS sequestration by cholestyramine has been observed under in vitro ruminal conditions [13]. On a related styrenic anion-exchange resin, low-concentration affinity increased with carboxylate chain length, and sulfonates showed greater affinity than carboxylates with the same number of perfluorinated carbons. Maximum capacity followed a different ordering, and PFOS suppressed carboxylate uptake in mixtures [14]. Resin hydration is also relevant. Calorimetry identified tightly bound water in cholestyramine and different heats of hydration for its chloride and nitrate forms [15]. Cholestyramine also binds bile acids [8], making competition with biological anions an important question for intestinal sequestration. Molecular exchange energetics can help explain these interactions alongside measurements of resin capacity and competitive uptake.

Here, we present a computational model of PFAS anion exchange with cholestyramine. Each cholestyramine model tests a different part of the exchange environment. BTMA^+^ isolates one quaternary ammonium site and tests local ion pairing. The extended monomer adds a divinylbenzene group and tests the effect of nonpolar resin contacts. Molecular dynamics simulations use a hydrated pVBTMA_12_ segment to test whether a prepared PFOA-associated state persists across separately seeded replicas. Density functional theory compares electronic and free energy trends for PFAS exchange. Energy-decomposition analysis and natural bond orbital analysis classify intermolecular and orbital contributions [16,17]. A constrained potential energy surface scan maps one set of local PFOA, chloride, and BTMA arrangements. We prioritized PFOS, PFOA, PFHxA, and 6:2 FTCA because these PFAS were detected in human autopsy tissues [18]. The results provide testable molecular hypotheses.

## 2. Methods and Materials

**Figure 1:**
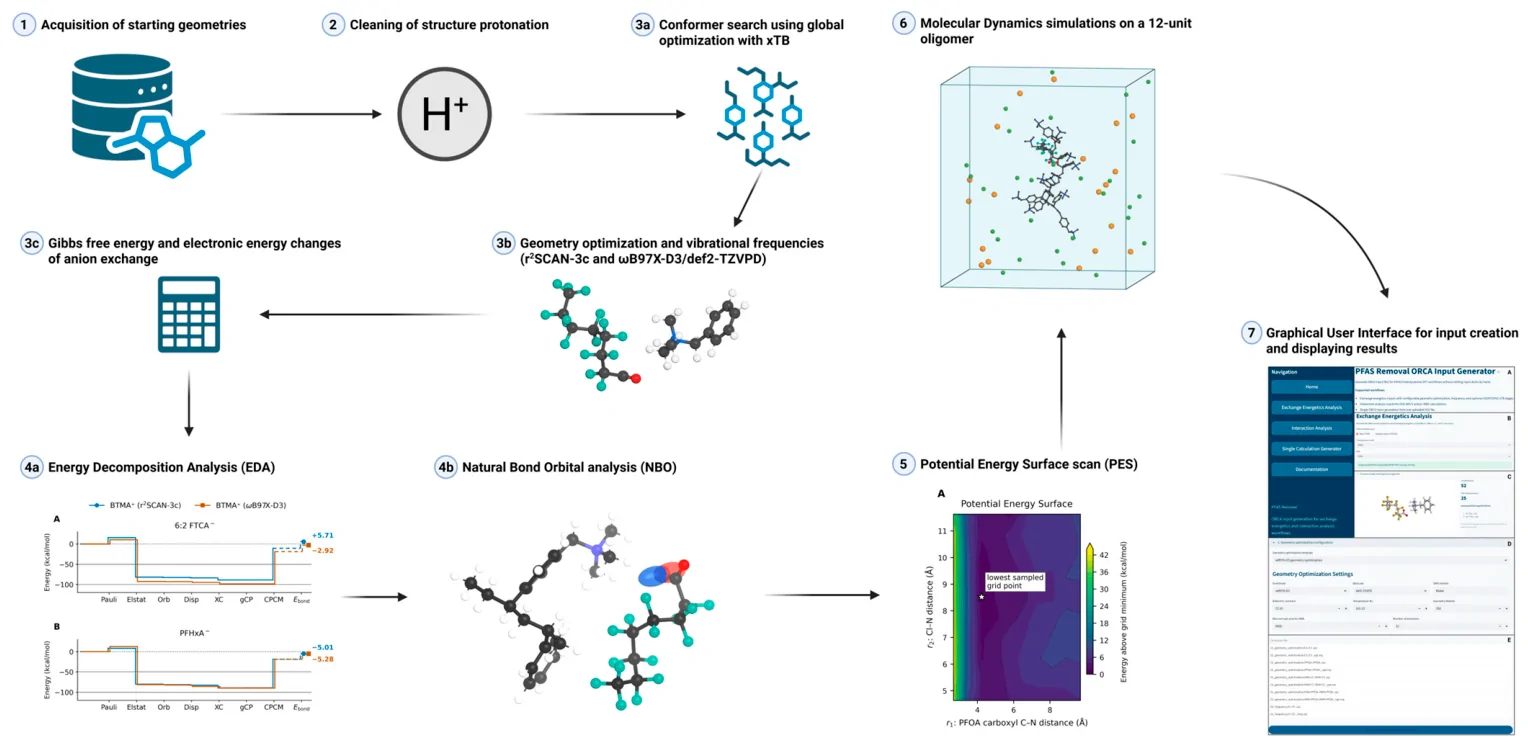
Workflow diagram of study methods. The major calculation and analysis steps are shown. Additional details for each workflow step are provided in the following sections. Created in BioRender (Science Suite Inc., Toronto, ON, Canada). Zwa, R. (2026) https://BioRender.com/z4gl4vs (accessed on 7 October 2026).

The computational workflow is summarized in the following workflow diagram (Figure 1). Implicit solvation with water was used for the BTMA and extended monomer calculations unless otherwise stated. The DVB-BTMA^+^PFOA^−^ and DVB-BTMA^+^Cl^−^ bound states were also evaluated with SMD/CPCM 1-octanol. Isolated anions remained aqueous.

### 2.1. Preparation of Relevant Structures

#### 2.1.1. Acquisition and Processing of PFAS and Cholestyramine Monomer Structures from PubChem

Initial molecular coordinates for the PFAS molecules and cholestyramine resin models were downloaded from PubChem as structured data files. Four PFAS were investigated because they have been detected in human autopsy tissues [18]. These molecules were perfluorooctanesulfonic acid (PFOS, CID 74483), perfluorooctanoic acid (PFOA, CID 9554), perfluorohexanoic acid (PFHxA, CID 67542), and 6:2 fluorotelomer carboxylic acid (6:2 FTCA, CID 11783764) [19,20,21,22]. In the deposited files, FHEA denotes 6:2 FTCA (CID 11783764).

Two reduced cholestyramine model structures were used to compare ammonium headgroup and backbone effects. The first model was benzyltrimethylammonium (BTMA, CID 5964) [23]. The BTMA^+^ cation has formula C_10_H_16_N^+^ and one quaternary ammonium center attached to a benzyl substituent. The second model was a styrene-divinylbenzene fragment with one benzyltrimethylammonium site (CID 22892408), referred to here as the extended monomer model [24]. DVB-BTMA^+^ denotes this extended monomer in complexes. The modeled cation has formula C_22_H_32_N^+^, formal charge +1, and one exchange site. Its connectivity is trimethyl[[4-(5-phenylhexan-3-yl)phenyl]methyl]azanium: a benzyltrimethylammonium group with a 5-phenylhexan-3-yl substituent para to the benzyl group. Atomic coordinates are provided with the deposited calculation files. PFAS complexes are denoted DVB-BTMA^+^P^−^, and the chloride reference is denoted DVB-BTMA^+^Cl^−^. Similar conventions are used for BTMA complexes. The extended model tests how added backbone contact and resin-like solvation affect exchange relative to BTMA. Both structures are reduced models and do not represent the complete crosslinked resin.

Protonation states of all investigated molecules were assigned according to a physiological pH of 7.4. This value was chosen because the ileum is the main location of PFAS enterohepatic recirculation [2]. The upper regions of the gastrointestinal tract, such as the jejunum, are more acidic (pH approximately 6.6). However, the pH within the ileum is about 7.4 [25]. The Open Babel plugin (version 3.1.1, Open Babel development team) within the Avogadro 2 software package (version 1.101.0-4, Open Chemistry project) was used to assign these states [26,27,28]. All PFAS were modeled in their fully deprotonated anionic forms. This modeling choice was justified by the low pK_a_ values of the sulfonic and carboxylic acid headgroups. These molecules remain anionic in the physiological pH range of the human body. The General AMBER Force Field (GAFF) was used in Avogadro to relax the initial coordinates [29]. This step reduced the cost of more computationally demanding methods by placing starting geometries near local potential energy minima. The coordinates generated by the GAFF method were thus used as inputs for later density functional theory and global optimization calculations. Molecular structures were rendered using open-source PyMOL (version 3.1.0; Schrödinger, LLC, New York, NY, USA).

#### 2.1.2. pVBTMA_12_ Polymer Segment Construction

The molecular dynamics model was a hydrogen-capped 12-unit poly(vinylbenzyltrimethylammonium) segment, denoted pVBTMA_12_. It has formula C_144_H_218_N_12_, formal charge +12, 374 atoms, 156 heavy atoms, 12 aromatic rings, and 12 quaternary ammonium sites. A deterministic Python workflow (version 3.12, Python Software Foundation, Beaverton, OR, USA) using RDKit (version 2023.09.3, RDKit development team) built the constitutional graph and explicit stereochemical definition from the polymerized repeat unit [30]. The selected stereoisomer contains 11 assigned backbone stereocenters. The terminal methylene carbon in repeat 12 is nonstereogenic.

Open Babel 3.1.1 generated and UFF-minimized the initial three-dimensional geometry [27,28]. RDKit checks confirmed the expected formula, formal charge, atom count, bond connectivity, ammonium coordination, aromatic ring count, and specified stereoisomer in the generated and minimized structures. pVBTMA_12_ is a finite linear segment. It was used to study local PFOA contacts and polymer rearrangement in explicit water. A larger crosslinked model would be required to represent the network structure of a complete resin bead.

### 2.2. Energetic and Thermodynamic Analysis of Anion Exchange Using the BTMA Model

#### 2.2.1. Screening of Anion Exchange with r^2^SCAN-3c and the BTMA Model

A screening study tested how PFAS identity affects anion exchange favorability in the BTMA model. For each PFAS, the exchange cycle included four species: the BTMA^+^P^−^ complex, the BTMA^+^Cl^−^ complex, the isolated PFAS anion (P^−^), and isolated chloride (Cl^−^). In this notation, P^−^ represents a PFAS anion. Each isolated PFAS anion was optimized and subjected to vibrational frequency analysis to obtain the thermochemical quantities required for Gibbs free energy calculations. The BTMA^+^Cl^−^ complex was treated using the same optimization and frequency workflow. The BTMA^+^P^−^ complexes were prepared by placing each PFAS anion near the cationic ammonium site of BTMA in Avogadro 2. The anionic headgroup was oriented toward the quaternary ammonium group. Geometry optimization and frequency analysis followed. Free chloride was included as the fourth species in the exchange cycle. Equation (1) was used to calculate the change in Gibbs free energy (Δ*G*_exchange_) for each anion exchange mechanism. Here, Ch^+^ represents a cationic cholestyramine model site, P^−^ represents a PFAS anion, and Cl^−^ represents chloride:

Δ*G*_exchange_ = *G*_Ch+P−_ − *G*_Ch+Cl−_ − *G*_P−_ + *G*_Cl−_ (1)

*G*_Ch+P−_ and *G*_Ch+Cl−_ refer to the Gibbs free energies of the PFAS anion and chloride in complex with the generic cholestyramine model site, and *G*_P−_ and *G*_Cl−_ refer to the Gibbs free energies of the isolated anions. The same formulation was used to calculate the electronic exchange energy (Δ*E*_exchange_). For Δ*E*_exchange_, the electronic energy of each species was taken from its optimized geometry at the stated level of theory. The four species were not evaluated on a common geometry. All vibrational modes were inspected for imaginary frequencies. This check was needed because calculated Δ*G*_exchange_ values require local minima, and imaginary modes indicate departure from a local minimum on the respective potential energy surface.

At this stage, all geometry optimizations and vibrational frequency calculations were performed using the r^2^SCAN-3c composite functional in the ORCA software suite (version 6.1.0, FACCTs GmbH, Cologne, Germany) [31,32]. This meta-GGA method was used because it gives accurate conformational energies and noncovalent interactions at lower computational cost than traditional functionals [33]. r^2^SCAN-3c was also used because of its custom basis set with specific corrections for basis set superposition error, dispersion forces via the D4 method, and basis set contraction effects. The Solvation Model based on Density (SMD) and the Conductor-like Polarizable Continuum Model (CPCM) were used for all DFT calculations [34,35]. Water was specified as the solvent to approximate the aqueous conditions of the ileum. A dielectric constant (*ε*) of 72.5 was used for the aqueous DFT calculations to represent the physiological conditions of the human ileum and simulate the dielectric decrement caused by 0.15 M NaCl at 310.15 K. This value was calculated by first using an exponential decay equation to determine the *ε* value of water at a body temperature of 310.15 K [36]. A linear dielectric decrement equation was then applied to account for the salt concentration of the gastrointestinal tract [37,38]. The ileum is considered isotonic with plasma, which has a sodium concentration between 137 and 142 mEq/L [39,40].

Numerical integration used the DefGrid3 setting to increase numerical precision. The resolution of identity with a chain of spheres exchange (RIJCOSX) approximation [41] was used to accelerate exchange and Coulomb integral evaluation. The def2/J basis set [42] was used for this approximation. Strict optimization criteria (tightopt) were enforced at all steps.

#### 2.2.2. Screening of Anion Exchange with ωB97X-D3 and the BTMA Model

Additional geometry optimizations and frequency calculations used the ωB97X-D3 range-separated hybrid functional [43]. This functional uses a varying percentage of Hartree-Fock exchange that depends on the distance between interacting electrons. Range separation reduces self-interaction errors, which can otherwise lead to inaccurate results for anionic systems. The ωB97X-D3 method also includes empirical dispersion corrections, which support its use for studying noncovalent interactions between PFAS and the resin backbone. The single polarized diffuse def2-TZVPD basis set [44,45] was used at this stage, alongside ωB97X-D3. The inclusion of diffuse functions may more accurately represent the expanded electron density of the anionic PFAS headgroups and the distributions of lone pairs.

The initial screening step used both the r^2^SCAN-3c and ωB97X-D3 functionals to calculate the thermodynamics of ion exchange, which permitted comparisons between the two methods. All procedures discussed in Section 2.2.1, including geometry optimization, electronic energy analysis, vibrational frequency analysis, and calculation of both Δ*E*_exchange_ and Δ*G*_exchange_, were used for calculations with ωB97X-D3, with the exception of the density functional and basis set.

#### 2.2.3. Analysis of Functional Dependence of Δ*E*_exchange_ and Δ*G*_exchange_ in the BTMA Model

The BTMA anion exchange energies were separated into electronic and thermochemical components. This separation showed how the results of r^2^SCAN-3c and ωB97X-D3 differed in the BTMA model. Quantities labeled with “exchange” refer to the full thermodynamic cycle defined in Equation (1), while those without this label correspond to an individual optimized structure. Each reported exchange value included contributions from the BTMA^+^P^−^ complex, the BTMA^+^Cl^−^ reference complex, the isolated PFAS anion, and isolated chloride. For a single species in the cycle, the thermochemical correction is defined as *G* − *E*_el_, where *E*_el_ is the electronic energy of that species, and *G* is the Gibbs free energy of that same species after zero-point, thermal, enthalpic, and entropic corrections are applied to the frequency calculation.

For the full exchange cycle involving a given PFAS anion, the corresponding thermochemical correction is written as Δ(*G* − *E*_el_)_exchange_. This term is the sum of the *G* − *E*_el_ corrections for each species in Equation (1):

(2) $$\Delta {\left(G-E_{\mathrm{el}}\right)}_{\mathrm{exchange}}={\left(G-E_{\mathrm{el}}\right)}_{\mathrm{Ch}+P-}-{\left(G-E_{\mathrm{el}}\right)}_{\mathrm{Ch}+\mathrm{Cl}-}-{\left(G-E_{\mathrm{el}}\right)}_{P-}+{\left(G-E_{\mathrm{el}}\right)}_{\mathrm{Cl}-}$$

The final exchange free energy can then be written as:

Δ*G*_exchange_=Δ*E*_exchange_+Δ(*G* − *E*_el_)_exchange_ (3)

A positive net thermochemical correction increases Δ*G*_exchange_ relative to Δ*E*_exchange_, while a smaller positive correction produces a lower final Δ*G*_exchange_ for the same electronic exchange energy.

Differences between functionals have been labeled using the term Δ_F_, which denotes the ωB97X-D3 value minus the r^2^SCAN-3c value for a given quantity. For example, Δ_F_Δ*E*_exchange_ refers to the difference between ωB97X-D3 and r^2^SCAN-3c in the value of Δ*E*_exchange_, while Δ_F_Δ(*G* − *E*_el_)_exchange_ refers to the difference between functionals in the net thermochemical correction. For Δ(*G* − *E*_el_)_exchange_, a positive Δ_F_Δ(*G* − *E*_el_)_exchange_ means that the range-separated hybrid estimates a higher thermochemical correction compared to the composite functional. For an individual species, Δ_F_Δ denotes its signed contribution to the functional difference in the exchange thermochemical correction. The difference between its two *G* − *E*_el_ values is multiplied by its coefficient in Equation (2). These signed contributions sum to the functional difference in the net exchange correction. For example, Δ_F_Δ${\left(G-E_{\mathrm{el}}\right)}_{\mathrm{Ch}+\mathrm{Cl}-}$ would be given by the expression [−(ωB97X-D3 [${\left(G-E_{\mathrm{el}}\right)}_{\mathrm{Ch}+\mathrm{Cl}-}$] − r^2^SCAN-3c [${\left(G-E_{\mathrm{el}}\right)}_{\mathrm{Ch}+\mathrm{Cl}-}$])] due to the species Ch^+^Cl^−^ carrying a negative sign in Equation (2).

### 2.3. Energetic and Thermodynamic Analysis of Anion Exchange Using the Extended Monomer Model

#### 2.3.1. Refinement of Exchange Thermodynamics Using Global Optimization of the Extended Monomer Model

The global optimizer algorithm (GOAT) [46] in ORCA searched for low-energy conformations of the starting structures used for the extended monomer calculations. These structures were the free PFAS anions, the unbound extended monomer, and the bound complexes for each DVB-BTMA^+^P^−^ system. Similar to the geometry optimization of the BTMA model, molecules were placed so that the anionic headgroup of the PFAS anion was near the cationic ammonium functional group of the monomer. The GOAT workflow proceeded with an initial geometry optimization to relax the starting structure, followed by an unconstrained conformer search.

The GFN2-xTB semiempirical method [47] was used in the global optimization process because of its lower computational cost. Solvation effects were incorporated using the analytical linearized Poisson-Boltzmann (ALPB) model [48] with water as the solvent. Conformers were ranked by their search-level electronic energies. The lowest-energy structure found for each exchange species was refined by DFT. The resulting optimized electronic and Gibbs energies were used in Equation (1). A common aqueous isolated-PFAS reference and chloride reference were used for both resin models at each functional, so model comparisons vary the bound species while holding the free anions fixed.

A conformer sensitivity test used the original GOAT candidate and two low-energy alternatives for each of DVB-BTMA^+^PFOA^−^, DVB-BTMA^+^Cl^−^, and isolated PFOA^−^. Their source ensemble indices and search energies are reported. Each candidate was optimized and subjected to an aqueous SMD/CPCM r^2^SCAN-3c frequency calculation at 310.15 K. Quasi-RRHO thermochemistry used a 100 cm^−1^ reference frequency and a separate 1 cm^−1^ low-frequency cutoff. Heavy-atom RMSD after rigid alignment was minimized over element- and connectivity-preserving atom permutations to compare the starting and refined geometries. For each alternative exchange value, one species was substituted while the other species used their original conformers. Additional conformer optimizations and frequency calculations used r^2^SCAN-3c because the corresponding ωB97X-D3 calculations were computationally more expensive.

#### 2.3.2. Comparison of Solvent Identities for Implicit Solvation of the Resin Environment

The solvent identity comparison tested the extended monomer PFOA exchange cycle with water and 1-octanol treatments of the two bound complexes, DVB-BTMA^+^PFOA^−^ and DVB-BTMA^+^Cl^−^. The isolated PFOA^−^ and Cl^−^ anions remained aqueous in both calculations. Both 1-octanol bound states were optimized and evaluated with SMD/CPCM 1-octanol and *ε* = 9.8629. Thus, the comparison varies the continuum treatment of the bound states while holding the isolated-anion references fixed. The true solvent structure around sites in hydrated cholestyramine remains uncertain.

### 2.4. Energy Decomposition and Analysis of Orbital Overlaps in Bound States

Energy-decomposition analysis with natural orbitals for chemical valence (EDA-NOCV) [16] was performed in ORCA 6.1.0 to identify binding energy components. For the BTMA model, the four BTMA^+^P^−^ complexes from both the r^2^SCAN-3c and ωB97X-D3 optimizations were used as EDA input structures. An identical EDA process was used for the four PFAS in the DVB-BTMA model. An additional EDA calculation was performed for the DVB-BTMA^+^PFOA^−^ complex using 1-octanol implicit solvation. Each complex was divided into two fragments. The first contained the cationic cholestyramine model (BTMA or extended monomer), and the second contained the anionic PFAS molecule. ORCA reports Pauli, electrostatic, orbital, dispersion, exchange-correlation, gCP where applicable, and CPCM dielectric terms separately. The directly reported Bond Energy is the complex electronic energy relative to the two fragments at the complex geometry. The listed components and this frozen-fragment endpoint were tabulated separately. The electrostatic term quantified the classical attraction between opposite charges. The Pauli term accounted for the destabilizing repulsion between occupied electron shells. The orbital term describes the stabilization from electron density transfer and polarization. The dispersion term was analyzed separately to determine the stabilization provided by van der Waals forces along the perfluoroalkyl chain.

NBO analysis used NBO (version 7.0.9, Theoretical Chemistry Institute, University of Wisconsin–Madison, Madison, WI, USA) [17] in ORCA 6.1.0 to study orbital interactions in the DVB-BTMA^+^PFOA^−^ complex. The initial conformer was identified using the GOAT global optimization procedure with GFN2-xTB and ALPB water solvation, followed by r^2^SCAN-3c optimization and frequency analysis in SMD water. The structure was then optimized using ωB97X-D3/def2-TZVPPD in SMD water. The final optimized geometry contained no imaginary frequencies. NBO analysis was performed as a ωB97X-D3/def2-TZVPD single-point calculation with TightSCF and CPCM/SMD water solvation. Natural population analysis, natural localized molecular orbital analysis, bond index analysis, canonical molecular orbital analysis, dipole analysis, and NBO archive output were requested. Second-order perturbation analysis was used to identify donor-acceptor interactions. Natural atomic orbital energies and occupancies were used to describe the orbitals associated with these interactions. NBO reported interatomic linear dependence in the diffuse basis and discarded several diffuse subshells during the analysis. Second-order stabilization energies on the order of 0.1–0.2 kcal/mol were interpreted qualitatively.

### 2.5. Constrained Potential Energy Surface Scan

A two-dimensional relaxed potential energy surface (PES) scan was performed to sample constrained local arrangements of PFOA, chloride, and BTMA. PES scanning was performed in ORCA using the semiempirical xTB method [47]. Solvation effects were simulated using the analytical linearized Poisson-Boltzmann (ALPB) model [48] with water as the solvent. The scanned coordinates were the chloride to ammonium nitrogen distance and the distance from the PFOA carboxyl carbon to the same nitrogen. A 10 by 10 grid was evaluated between the specified coordinate endpoints. The two target distances were constrained at each grid point while all remaining internal degrees of freedom were optimized, giving 100 geometries.

Total electronic energies for the 2D grid were extracted from structure files for each scanned step, which are available in the Zenodo archive at https://doi.org/10.5281/zenodo.23087140 (accessed on 1 October 2026). The data were processed into a regular grid based on the unique values of the scanned bond lengths. Relative energies were computed by subtracting the lowest sampled grid energy from all grid points. These relative energies were used to generate contour and surface representations of the constrained grid.

### 2.6. Molecular Dynamics Simulations

Molecular dynamics simulations examined PFOA contact and oligomer rearrangement in a hydrated pVBTMA_12_ system. Three 10 ns production continuations used common prepared coordinates and separate velocity seeds.

#### 2.6.1. Polymer Model and Force Field Construction

pVBTMA_12_ was parameterized using AmberTools (version 25.3.0, Amber development team) with General AMBER Force Field 2 (GAFF2) atom types [49]. A five-unit capped pVBTMA model was used for the AM1-BCC charge calculation [50]. RDKit 2023.09.3 performed atom-role mapping and generated a seeded ETKDGv3 starting conformer, followed by UFF minimization before SQM/AM1-BCC calculations [30]. Charges were separated into head, internal, and tail repeat libraries. Each repeat library was normalized to +1 before transfer to the 12-unit chain. The transferred pVBTMA_12_ charges summed to +12 before serialization.

Automated mapping checks confirmed complete atom coverage and the expected repeat assignments. Full-chain connectivity checks confirmed 374 atoms, 385 bonds, and 12 four-coordinate nitrogen atoms. A full-chain parmchk2 assessment found no flagged missing GAFF2 terms. Topology validation used ParmEd (version 4.3.0, ParmEd development team). A dry LEaP topology reproduced the source bond indices and gave a charge of +11.99999404 e, within the 10^−5^ e tolerance. The serialized MOL2 sum was +11.999994 e. Its 6 × 10^−6^ e residual exceeded the strict 10^−6^ e test but was within the documented serialization bound. Transferred charges agreed with direct whole-chain AM1-BCC charges within the predefined per-group RMSE and maximum-difference limits of 0.010 and 0.040 e, respectively. Deprotonated PFOA used GAFF2 atom types and AM1-BCC charges with net charge −1. Standard AMBER parameters were used for sodium, chloride, and TIP3P water [51,52].

#### 2.6.2. Solvated System Construction

The chloride-form pVBTMA_12_ system was placed in a 15 Å TIP3P solvent buffer. Twelve chloride ions neutralized the polymer, and 19 sodium chloride pairs gave a nominal added-salt concentration of 0.154 M. The accepted chloride-form endpoint was then used to prepare one PFOA-associated state. PFOA was placed near repeat 9, with an initial nearest carboxylate oxygen to ammonium nitrogen distance of 4.43 Å. One distant chloride was removed only to preserve system neutrality, and 20 overlapping waters were removed. The final system contained one pVBTMA_12_, one PFOA, 19 sodium ions, 30 chloride ions, and 5163 waters. The system was neutral and contained 15,937 atoms. Composition, ion counts, periodic box, and water checks passed for the chloride-form precursor.

#### 2.6.3. Molecular Dynamics Protocol

All simulations used NAMD (Git-2025-12-04, Linux-x86_64-multicore-CUDA build with CUDA 11.8, University of Illinois Urbana-Champaign, Urbana, IL, USA) with AMBER topology and coordinate input [53]. Periodic electrostatics used particle mesh Ewald [54]. Nonbonded interactions used a 9 Å cutoff, an 11 Å pairlist, and the Lennard-Jones long-range correction. Bonds involving hydrogen were constrained, and the time step was 2 fs. Temperature was maintained at 310.15 K by Langevin dynamics with 1 ps^−1^ damping. NPT sampling used a Langevin piston at 1.01325 bar, with a 100 fs period and 50 fs decay [55]. No positional, terminal, shape, fixed-atom, extra-bond, or collective variable restraints were applied.

The accepted PFOA-associated pilot endpoint supplied common coordinates and cell dimensions for all replicas. Each replica received a distinct velocity seed, followed by 0.5 ns of NPT equilibration and ten restartable 1 ns NPT production segments. The production duration was 10 ns per replica. Coordinates were saved every 2 ps, giving 5000 production frames per replica. Pilot trajectories were used for preparation and acceptance only and were not included in the production statistics.

#### 2.6.4. Structural and Periodic Boundary Analysis

CPPTRAJ (version 6.29.13, AmberTools) autoimaging and explicit unit-cell shape handling were applied before distance and shape measurements. VMD (version 2.0.1a1, University of Illinois Urbana-Champaign, Urbana, IL, USA) with its PBCTools plugin (version 2.8) provided an independent check of the trajectory cell series. All three 5000-frame trajectory series passed both checks. The largest difference between DCD and XST cell lengths was approximately 2.4 × 10^−6^ Å.

The continuous metrics were polymer heavy-atom RMSD, mass-weighted radius of gyration, end-to-end distance, the nearest PFOA carboxylate oxygen to any polymer ammonium nitrogen, and the minimum PFOA-to-polymer heavy-atom distance. The nearest distance to the initially selected ammonium site was retained as a site-specific descriptor. Ammonium contact occupancy was the fraction of frames with either carboxylate oxygen at a distance of less than 5 Å from any ammonium nitrogen. Fluorinated-tail contact counted unique oligomer heavy atoms within 4 Å of the PFOA tail, and tail hydration counted waters within 5 Å of that tail.

#### 2.6.5. Statistical Analysis

Each 10 ns production trajectory was divided into ten contiguous 1 ns blocks to summarize temporal changes. Means and standard deviations were calculated separately for each replica. Integrated autocorrelation times were estimated from the full 2 ps series by summing normalized autocorrelation terms until the first nonpositive term. The convention included half the zero-lag contribution. Effective sample size was the frame count divided by twice the integrated autocorrelation time expressed in frames. Temporal sampling was screened by an effective sample size of at least 20 and a first-to-last 1 ns shift no greater than 0.5 within-replica standard deviations. Cross-replica spread was screened against one pooled within-replica standard deviation. These criteria assessed sampling of individual observables [56].

The plotted trajectories use consecutive, nonoverlapping 0.25 ns averages for all six observables. Each display window contains 125 frames, giving 40 means per replica. These plotting windows are separate from the 1 ns statistical summaries. The original correlated series supplied the autocorrelation and effective sample size estimates. Display-window counts were not used as independent sample sizes.

### 2.7. Development of GUI for ORCA Calculation Parameterization

**Figure 2:**
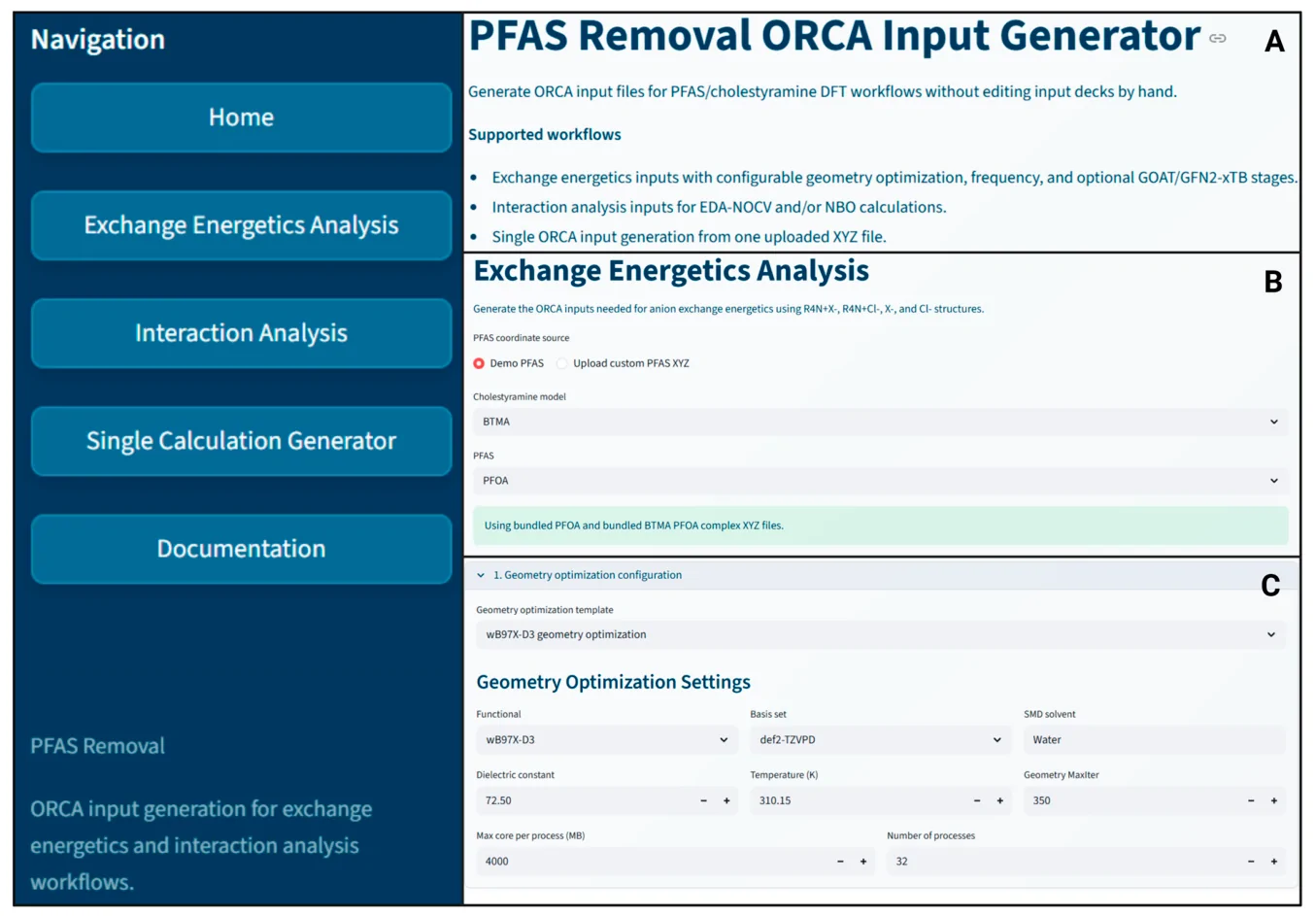
PFAS Removal graphical user interface for ORCA input generation. (**A**) Home view with navigation and available workflows. (**B**) Exchange energetics setup with example BTMA/PFOA selections. (**C**) Geometry optimization settings showing an SMD water example and selectable computational parameters. Created in BioRender. Zwa, R. (2026) https://BioRender.com/4a90wlm (accessed on 1 October 2026).

A web-based graphical user interface (GUI) was developed in Python 3.12 using Streamlit (version 1.45.1, Snowflake Inc., Menlo Park, CA, USA) to generate ORCA input files for the computational workflow. Users select a PFAS or upload its coordinates, choose the BTMA or extended monomer model, and set the functional, basis set, solvent, and computational resources. The full workflow generates inputs for GOAT/GFN2-xTB, DFT optimization and frequencies, EDA-NOCV, and NBO calculations. A separate mode generates an input for one selected calculation. The interface and representative settings are shown (Figure 2A–C).

The GUI exposes the dielectric constant and temperature alongside the solvent and electronic-structure settings. Inputs can therefore reproduce the specified water or 1-octanol protocols. The application is freely available at https://pfas-removal.streamlit.app/ (accessed on 1 October 2026), with source code and complete user documentation archived in Zenodo at https://doi.org/10.5281/zenodo.23087140 (accessed on 1 October 2026).

## 3. Results

**Figure 3:**
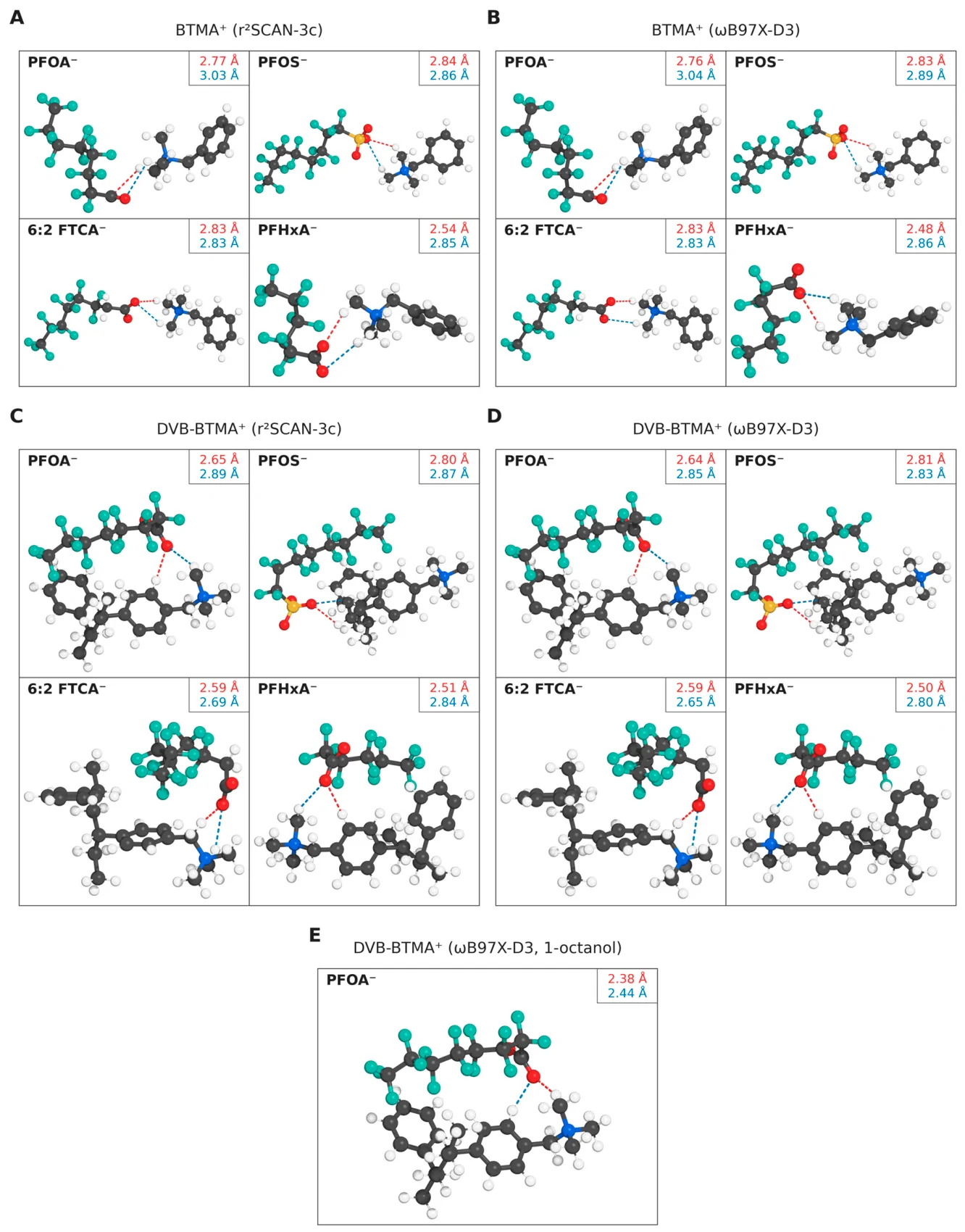
Optimized DFT ion-pair structures. (**A**,**B**) BTMA^+^ complexes at r^2^SCAN-3c and ωB97X-D3. (**C**,**D**) Extended DVB-BTMA^+^ complexes at the same levels. (**E**) DVB-BTMA^+^·PFOA^−^ structure in SMD/CPCM 1-octanol at ωB97X-D3. Water is the implicit solvent for (**A**–**D**). Carbon, hydrogen, nitrogen, oxygen, fluorine, and sulfur atoms are shown in dark gray, white, blue, red, teal, and yellow, respectively. Red and blue distance labels correspond to the contacts shown in the same colors. Dashed contacts show the displayed distances in Å.

The principal DFT exchange energies are summarized (Table 1). The optimized ion-pair structures are shown (Figure 3A–E).

### 3.1. Energetic and Thermodynamic Analysis of Anion Exchange in the BTMA Model

#### 3.1.1. Comparison of Δ*E*_exchange_ Between ωB97X-D3 and r^2^SCAN-3c for the BTMA Anion Exchange Model

**Figure 4:**
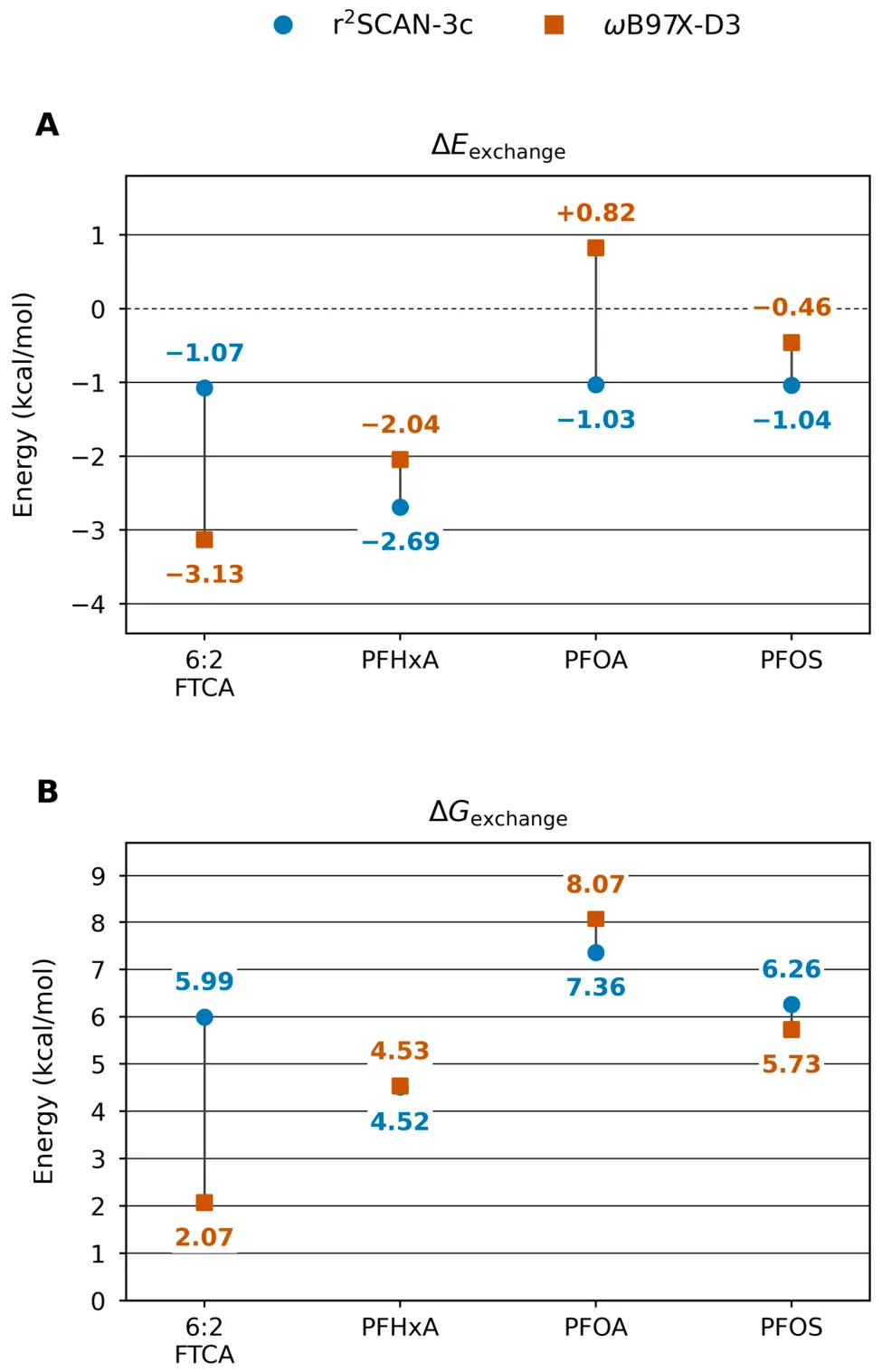
BTMA anion exchange energies. (**A**) Electronic exchange energy and (**B**) Gibbs free energy for the four PFAS at r^2^SCAN-3c and ωB97X-D3. Values are in kcal/mol at 310.15 K.

Electronic exchange energies for BTMA ranged from −2.69 to −1.03 kcal/mol at r^2^SCAN-3c and from −3.13 to +0.82 kcal/mol at ωB97X-D3. PFHxA gave the most negative value at r^2^SCAN-3c, whereas 6:2 FTCA gave the most negative value at ωB97X-D3. PFOA was the only positive electronic exchange at the hybrid level (Figure 4A).

#### 3.1.2. Comparison of Δ*G*_exchange_ Between ωB97X-D3 and r^2^SCAN-3c for the BTMA Anion Exchange Model

Gibbs free energies of exchange for BTMA were positive for all four PFAS at both functionals. They ranged from +4.52 to +7.36 kcal/mol at r^2^SCAN-3c and from +2.07 to +8.07 kcal/mol at ωB97X-D3. PFOA gave the highest value at both levels. PFHxA gave the lowest value at r^2^SCAN-3c, while 6:2 FTCA gave the lowest value at ωB97X-D3 (Figure 4B).

Compared with r^2^SCAN-3c, ωB97X-D3 gave lower exchange free energies for 6:2 FTCA and PFOS and a higher value for PFOA. The PFHxA difference was only +0.02 kcal/mol. The underlying source energies and exchange quantities are provided (Table S1a–c). Optimized BTMA^+^P^−^ structures are shown for both functionals (Figure 3A,B).

#### 3.1.3. Thermochemical Decomposition of Functional Dependence in BTMA Exchange Energetics

**Figure 5:**
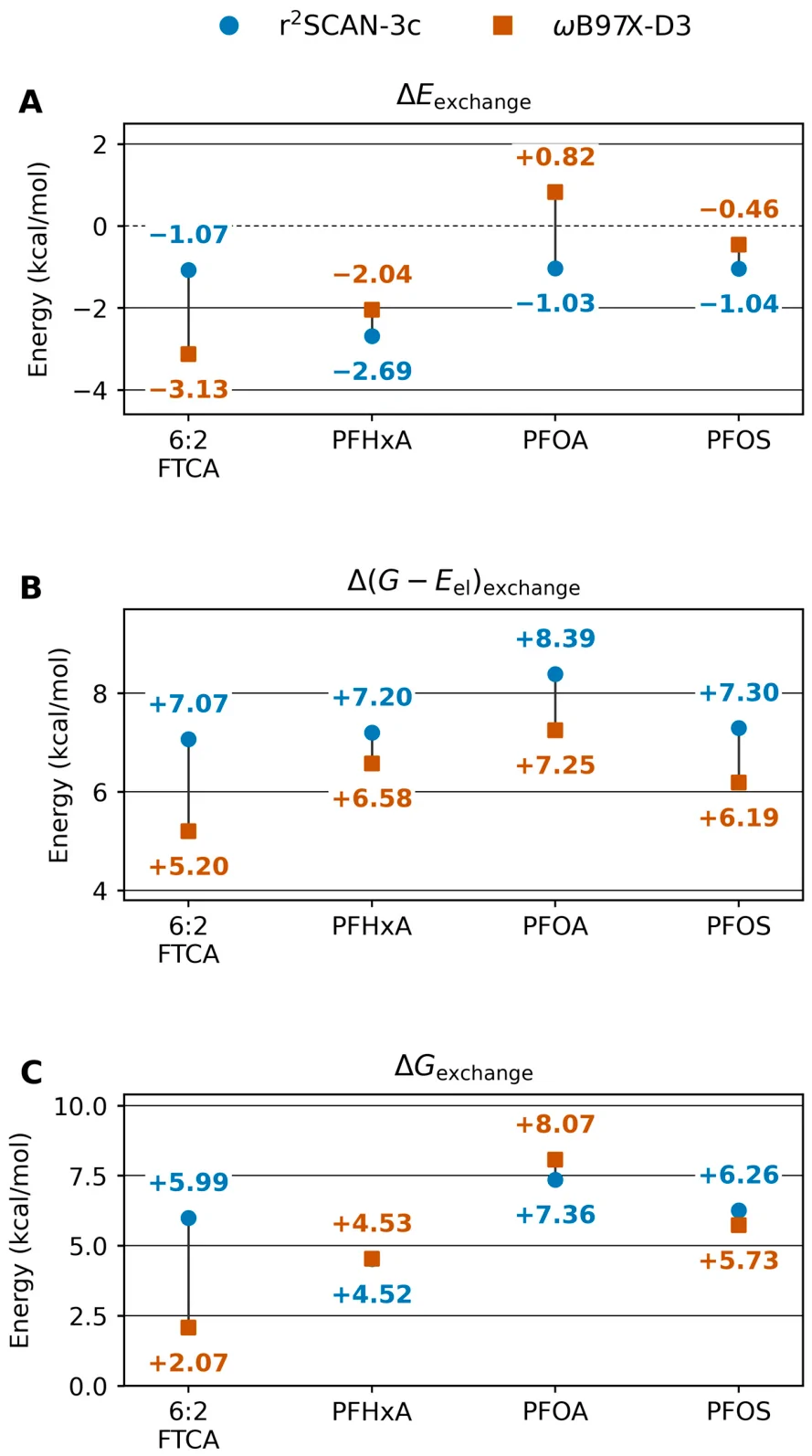
BTMA exchange energetics and thermochemical corrections. (**A**) Electronic exchange energies, (**B**) net thermochemical corrections, and (**C**) Gibbs exchange free energies for four PFAS. Paired points compare r^2^SCAN-3c and ωB97X-D3 at 310.15 K. Values are in kcal/mol.

**Figure 6:**
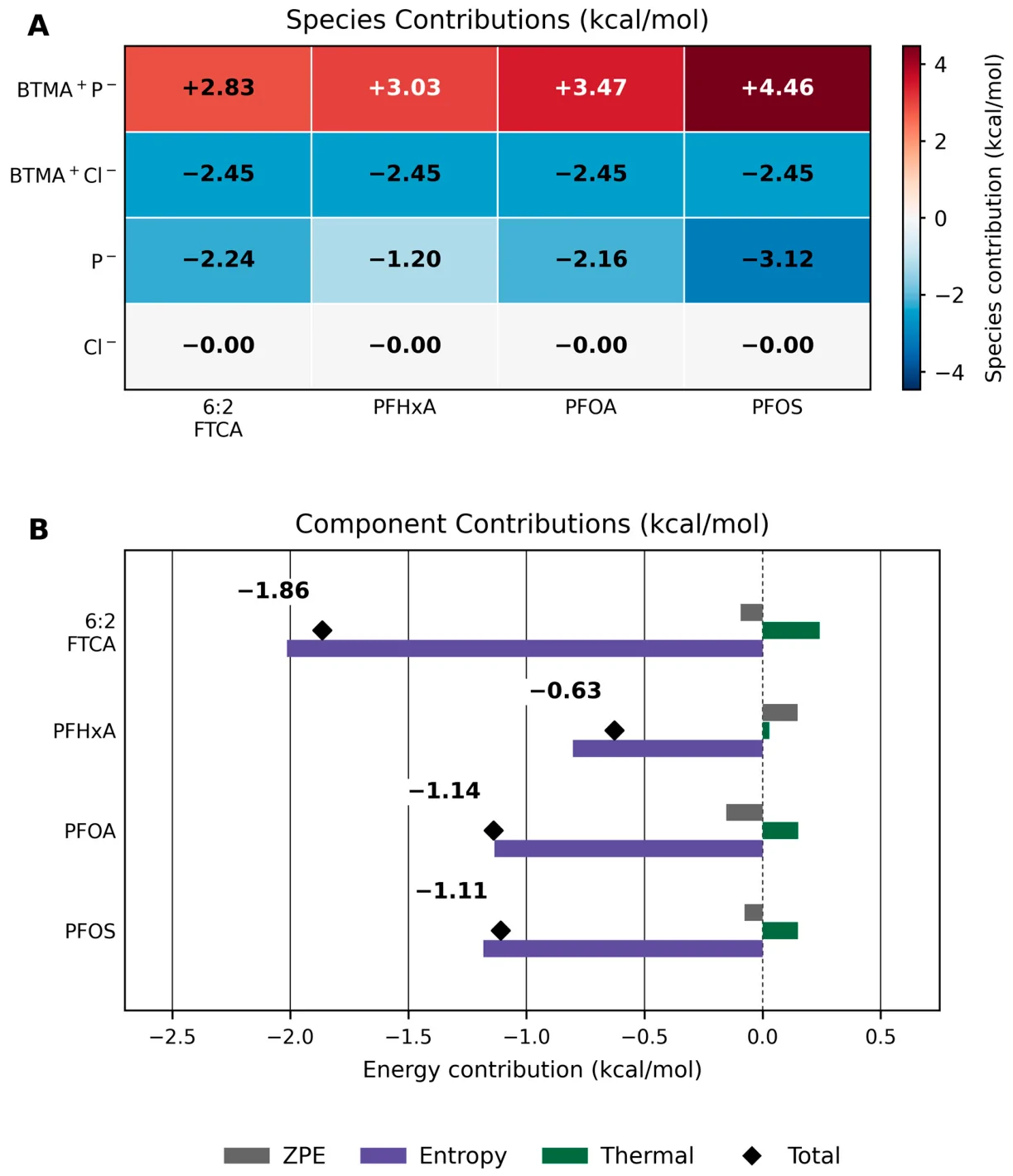
Functional dependence of BTMA thermochemical corrections. (**A**) Signed species contributions to the difference between ωB97X-D3 and r^2^SCAN-3c. (**B**) Zero-point, entropy, and finite-temperature contributions. Values are in kcal/mol.

ωB97X-D3 gave a more negative electronic exchange energy for 6:2 FTCA, while r^2^SCAN-3c gave more negative values for the other three PFAS. The net thermochemical correction was smaller at ωB97X-D3 for all four compounds. These corrections lowered the hybrid Gibbs exchange values for 6:2 FTCA and PFOS, but the electronic differences left PFOA higher and PFHxA nearly unchanged (Figure 5A–C and Figure 6A,B).

The contributions of each species to the functional difference in Δ(*G* − *E*_el_)_exchange_ were separated using the four species in Equation (2) (Figure 6A). Each heatmap cell represents the difference between functionals in *G* − *E*_el_ for each species, before application of the exchange-cycle coefficient. The arithmetic sign for each species in Equation (2) was applied to each value. The BTMA^+^P^−^ complex contribution was positive for all four PFAS, increasing from 6:2 FTCA to PFOS. The BTMA^+^Cl^−^ contribution was constant and negative for all four anion exchange reactions. The free PFAS anion contribution was negative for all PFAS and varied by structure, with the largest magnitude observed for PFOS. The free chloride contribution was negligible at the displayed precision for all four cycles.

The functional dependence of the net thermochemical correction, Δ_F_[Δ(*G* − *E*_el_)_exchange_], was separated into zero-point energy (ZPE), finite-temperature thermal correction, and entropy (Figure 6B). These values were calculated by applying Equation (2) to each component separately and then taking the functional difference.

#### 3.1.4. Energy-Decomposition Analysis of BTMA^+^P^−^ Complexes

**Figure 7:**
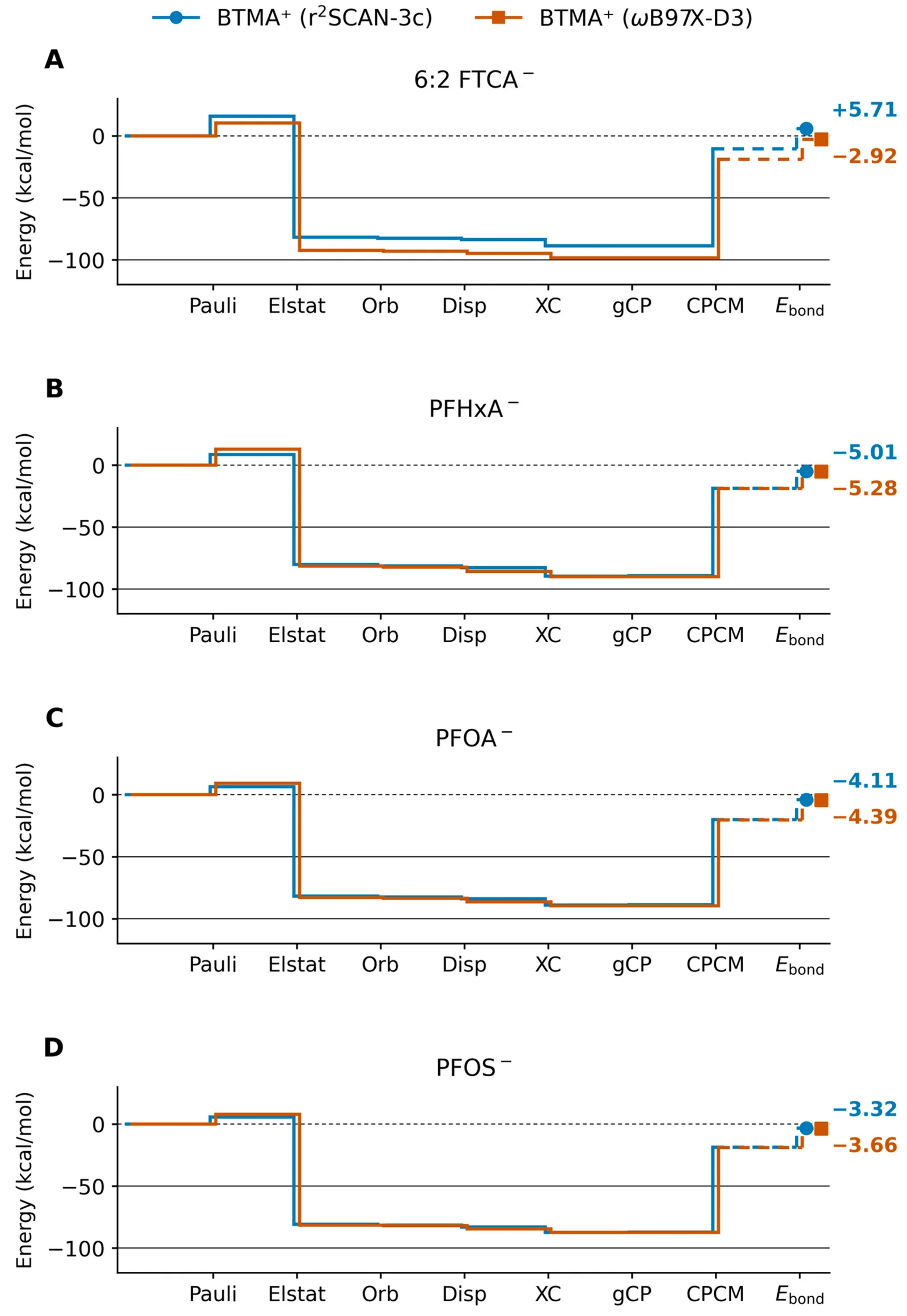
Energy-decomposition analysis of BTMA^+^P^−^ ion pairs in CPCM/SMD water. (**A**) 6:2 FTCA, (**B**) PFHxA, (**C**) PFOA, and (**D**) PFOS. The series compare r^2^SCAN-3c and ωB97X-D3. Solid steps show the listed components. Dashed segments connect their sum to the ORCA Bond Energy. Values are in kcal/mol (Table 2 and Table S2).

Energy-decomposition analysis of the BTMA^+^P^−^ complexes showed a similar term order for all four PFAS and both functionals (Figure 7A–D, Table 2 and Table S2). In each case, the most negative contribution was the electrostatic term. The CPCM dielectric term was the largest positive displayed contribution. Pauli repulsion also opposed electrostatic attraction. The orbital, dispersion, and exchange-correlation terms were smaller and consistently negative. The printed r^2^SCAN-3c gCP corrections were small.

For the BTMA^+^6:2 FTCA^−^ complex, the r^2^SCAN-3c decomposition gave a positive bond energy of +5.7 kcal/mol. The ωB97X-D3 decomposition had a bond energy of −2.9 kcal/mol (Figure 7A). The largest difference between the two functionals was observed in the Pauli term, which was smaller for ωB97X-D3. The electrostatic term was slightly more negative, and the dispersion term was also more stabilizing at the ωB97X-D3 level. For BTMA^+^PFHxA^−^, both decompositions resulted in similar negative bond energies at −5.0 kcal/mol for r^2^SCAN-3c and −5.3 kcal/mol for ωB97X-D3 (Figure 7B). Relative to r^2^SCAN-3c, the ωB97X-D3 results showed more Pauli repulsion and more negative electrostatic interaction. The dispersion term was also more negative for the range-separated hybrid.

For BTMA^+^PFOA^−^, the final bond energies were −4.1 kcal/mol for r^2^SCAN-3c and −4.4 kcal/mol for ωB97X-D3 (Figure 7C). The electrostatic and dispersion terms were again more negative for ωB97X-D3. The orbital and exchange-correlation terms were slightly less negative. The solvent correction remained one of the largest positive contributions in both decompositions. For BTMA^+^PFOS^−^, both functionals yielded negative bond energies at −3.3 kcal/mol for r^2^SCAN-3c and −3.7 kcal/mol for ωB97X-D3 (Figure 7D). The electrostatic term remained the largest negative contribution in both decompositions. The Pauli terms were positive and of comparable magnitude to those observed for PFOA. As in the other BTMA complexes, the dispersion contribution was more negative at the ωB97X-D3 level. The exchange-correlation term was less negative than in the corresponding r^2^SCAN-3c decomposition.

The listed EDA components did not sum to the reported Bond Energy. Preparation energy was not reported in these ORCA outputs, so the difference between Bond Energy and the component sum was not equated with preparation energy. The component sums and arithmetic differences are reported separately (Table S2).

### 3.2. Energetic and Thermodynamic Analysis of Anion Exchange in the Extended Monomer Model

#### 3.2.1. NBO Analysis of Orbital Interactions in the DVB-BTMA^+^PFOA^−^ Complex

**Figure 8:**
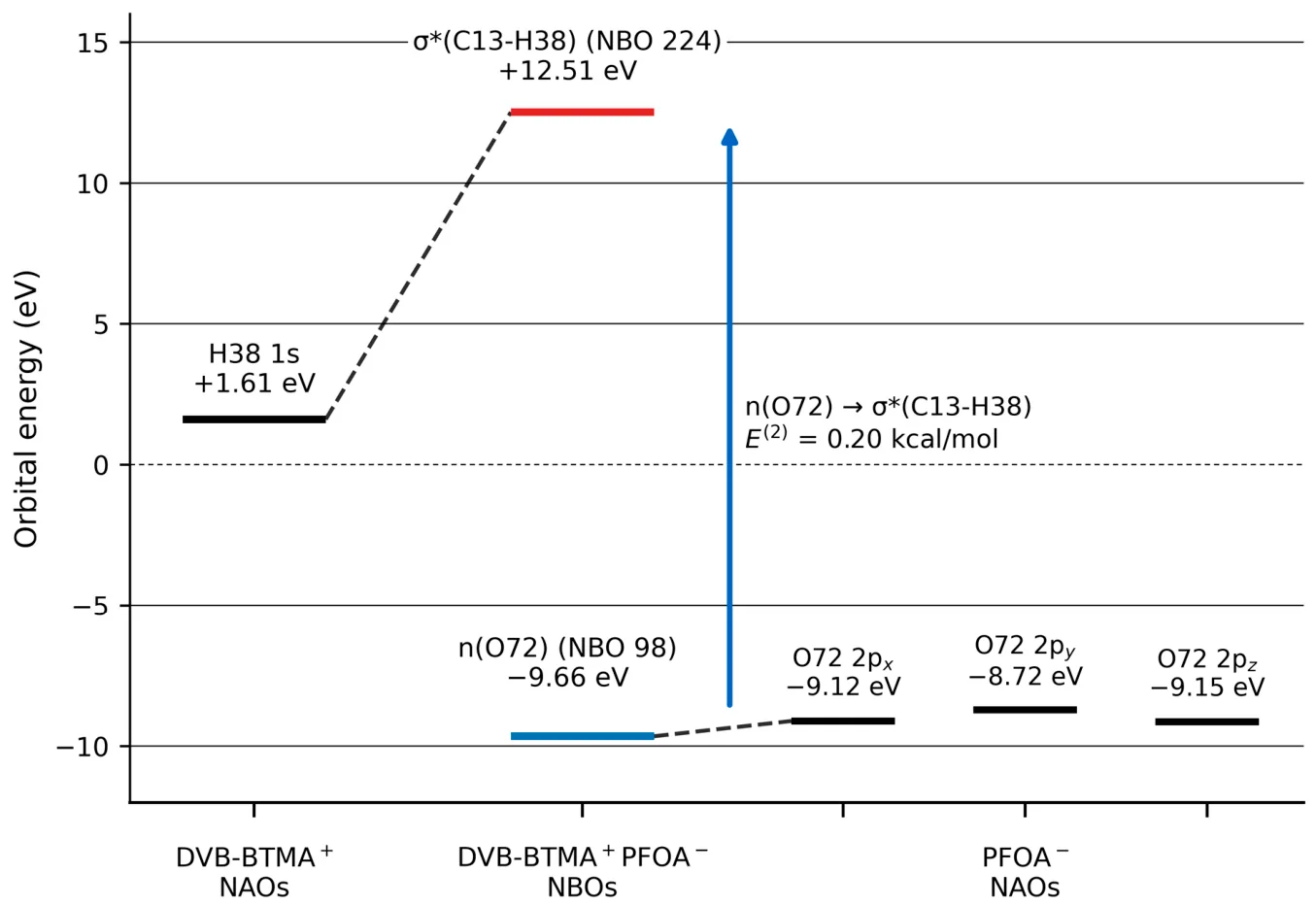
NAO-NBO energy-level diagram for the DVB-BTMA^+^PFOA^−^ complex. Black outer levels are NAO diagonal Fock matrix energies from the full complex. Central levels are localized NBO diagonal energies. The blue central level represents the O72 lone-pair donor, and the red central level represents the σ*(C13-H38) acceptor. Black dashed lines show orbital correlations. The LP(2) O72 → σ*(C13-H38) donor-acceptor delocalization with *E*^(2)^ = 0.20 kcal/mol is shown. The blue arrow identifies donor-to-acceptor delocalization. Its length does not represent *E*^(2)^, which is a perturbative stabilization estimate. Orbital energies are in eV.

NBO analysis of the DVB-BTMA^+^PFOA^−^ complex identified a donor-acceptor interaction from the LP(2) lone pair on PFOA oxygen O72 into the σ*(C13-H38) antibonding orbital of DVB-BTMA^+^ (Figure 8). The donor orbital, NBO 98, had an occupancy of 1.88 e and an energy of −9.66 eV. NBO 98 was composed of approximately 0.04% s, 99.78% p, 0.17% d, and 0.01% f character on O72. The acceptor orbital, NBO 224, had an occupancy of 0.00834 e and an energy of +12.51 eV. NBO 224 contained 37.67% C13 and 62.33% H38 character. The O72 2p_x_, 2p_y_, and 2p_z_ natural atomic orbitals were located at −9.12, −8.72, and −9.15 eV, respectively, while the H38 1s natural atomic orbital was located at +1.61 eV. The highlighted LP(2) O72 → σ*(C13-H38) interaction had an *E*^(2)^ stabilization energy of 0.20 kcal/mol (Figure 8).

#### 3.2.2. Comparison of Δ*E*_exchange_ Between BTMA and Extended Monomer Models at the ωB97X-D3 Level

**Figure 9:**
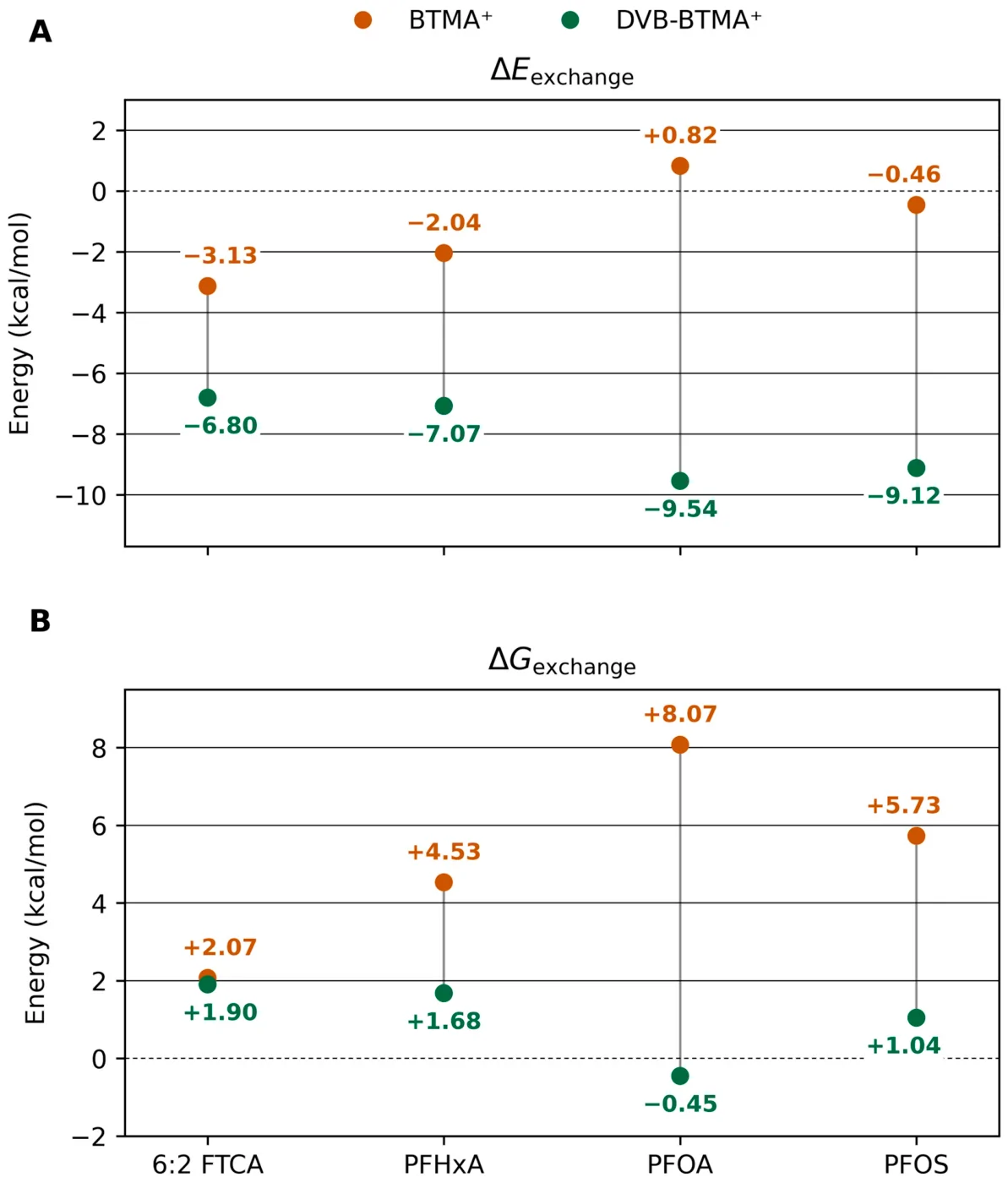
Effect of cholestyramine model size on anion exchange energetics. (**A**) Electronic exchange energy and (**B**) Gibbs exchange free energy for 6:2 FTCA, PFHxA, PFOA, and PFOS in the BTMA and extended monomer models at ωB97X-D3 with water implicit solvation. Values are in kcal/mol at 310.15 K.

The BTMA and extended monomer models were compared at the ωB97X-D3 level (Figure 9A, Table S3a–c). For all four PFAS, the extended monomer model gave more negative Δ*E*_exchange_ values than the corresponding BTMA model. The largest shift between models was observed for PFOA, at −10.37 kcal/mol. PFOA showed the most favorable Δ*E*_exchange_ in the extended monomer model, followed closely by PFOS.

#### 3.2.3. Comparison of Δ*G*_exchange_ Between BTMA and Extended Monomer Models at the ωB97X-D3 Level

With common aqueous free-anion references, the extended monomer lowered Gibbs exchange free energies relative to BTMA by 0.17, 2.85, 8.53, and 4.68 kcal/mol for 6:2 FTCA, PFHxA, PFOA, and PFOS, respectively. The 6:2 FTCA free energy was nearly unchanged by model expansion (Figure 9B).

The ordering of exchange free energies changed between the two models. In BTMA, 6:2 FTCA had the lowest hybrid value and PFOA the highest. In DVB-BTMA^+^, PFOA gave the lowest value, −0.45 kcal/mol, followed by PFOS, PFHxA, and 6:2 FTCA. The small negative PFOA value lies close to thermoneutral exchange. The optimized extended-model structures are shown for both functionals (Figure 3C,D).

#### 3.2.4. Effect of Implicit Solvent Identity on Extended Monomer Anion Exchange

**Figure 10:**
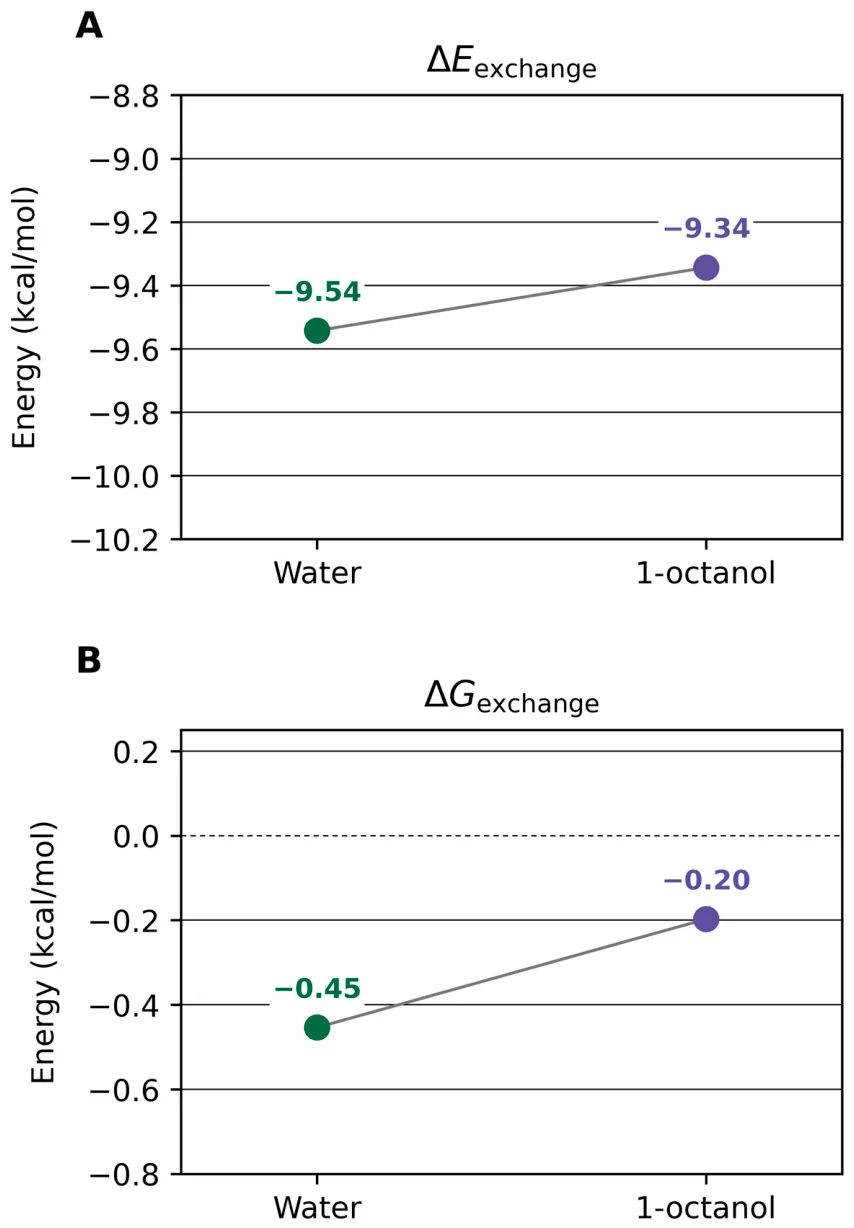
PFOA extended monomer exchange with two bound-state solvents. (**A**) Electronic and (**B**) Gibbs exchange energies for water and SMD/CPCM 1-octanol (*ε* = 9.8629). Isolated anions are aqueous in both cycles. Values are in kcal/mol.

Aqueous and 1-octanol bound-state calculations were compared for PFOA, while isolated anions remained aqueous. The electronic exchange energy shifted from −9.54 kcal/mol in water to −9.34 kcal/mol in 1-octanol (Figure 10A, Table S3c).

The PFOA Gibbs exchange energy shifted from −0.45 kcal/mol in water to −0.20 kcal/mol in 1-octanol, a change of +0.26 kcal/mol based on the unrounded energies (Figure 10B, Table S3c). The corresponding optimized structures are shown (Figure 3D,E). Extended monomer electronic energies and anion exchange energetics are reported in the Supplementary Tables (Table S3).

#### 3.2.5. Conformer Sensitivity of the PFOA Exchange Cycle

The aqueous r^2^SCAN-3c PFOA cycle gave Δ*E*_exchange_ = −9.32 and Δ*G*_exchange_ = +0.41 kcal/mol with the original candidates. Substituting the alternative PFOA complexes gave Gibbs exchange values of +0.41 and +0.67 kcal/mol. Alternative chloride complexes gave +0.80 and +0.65 kcal/mol. Isolated-PFOA alternatives gave −1.71 and −0.32 kcal/mol because their Gibbs energies were 2.12 and 0.73 kcal/mol higher than the original reactant. Thus, the negative substitutions arise from a higher-energy free reactant rather than a lower-energy bound product. Using the lowest tested Gibbs energy for each species gives +0.80 kcal/mol as an illustrative reconstruction of the tested states (Table S4a).

The refined PFOA-bound alternatives were nearly identical to the original, with symmetry-aware heavy-atom RMSDs of 0.007 and 0.054 Å. The chloride-bound and isolated-PFOA alternatives remained more distinct. The tested bound candidates therefore provide limited exploration of different final conformations. The substitutions describe selected-state sensitivity rather than conformer populations (Table S4b).

#### 3.2.6. Energy-Decomposition Analysis of DVB-BTMA^+^P^−^ Complexes

**Figure 11:**
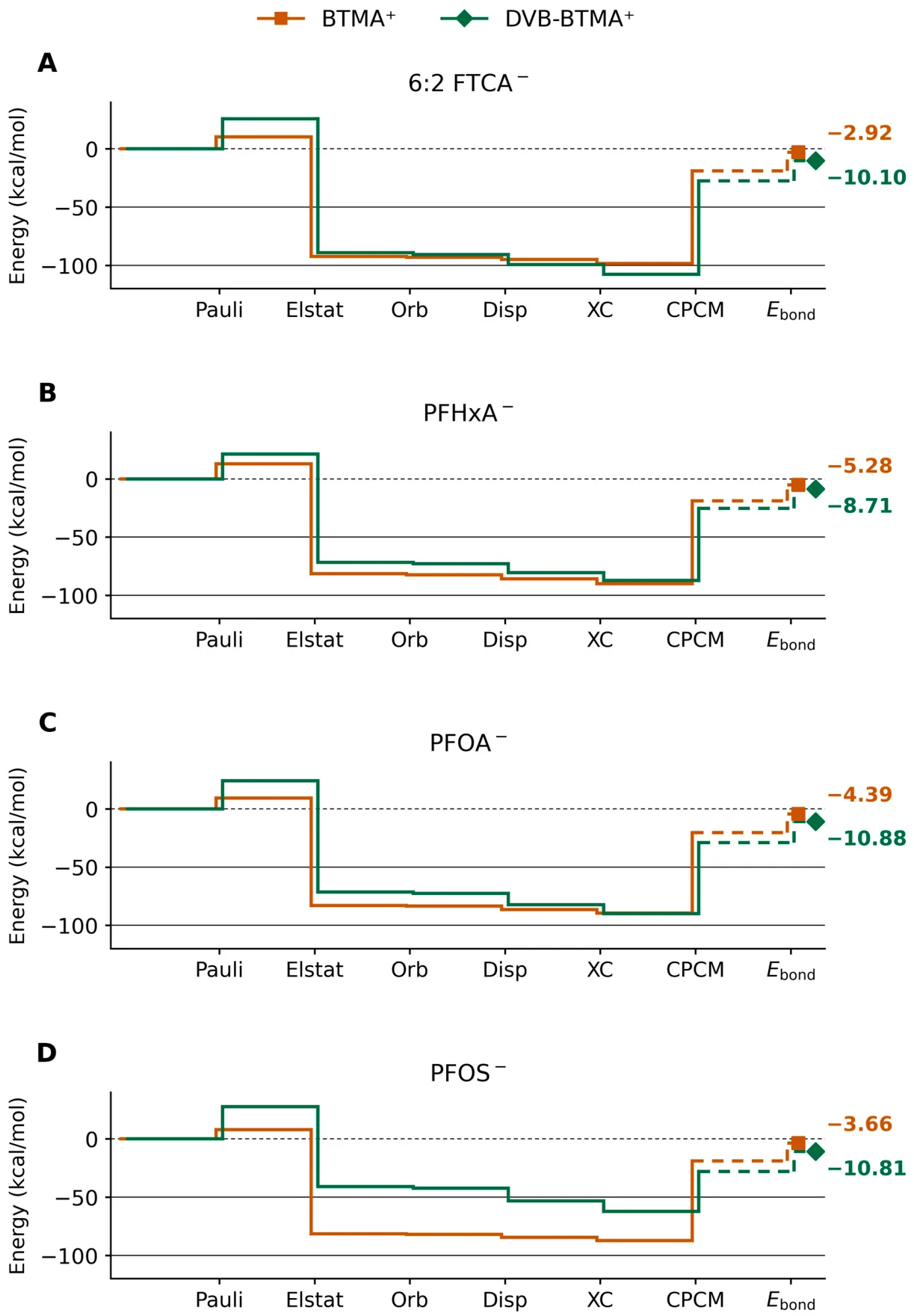
Energy-decomposition analysis of BTMA^+^ and DVB-BTMA^+^ ion pairs at ωB97X-D3/def2-TZVPD with CPCM/SMD water. (**A**) 6:2 FTCA, (**B**) PFHxA, (**C**) PFOA, and (**D**) PFOS. Solid steps show the listed components. Dashed segments connect their sum to the ORCA Bond Energy. Values are in kcal/mol (Table 2 and Table S2).

Energy-decomposition analysis compared the DVB-BTMA^+^P^−^ complexes with the BTMA model at the ωB97X-D3 level (Figure 11A–D). The electrostatic term was the largest negative contribution for all DVB-BTMA^+^P^−^ complexes. The extended monomer model gave more negative final bond energies for all four PFAS (Figure 11A–D). For DVB-BTMA^+^6:2 FTCA^−^, the final bond energy shifted from −2.9 kcal/mol in the BTMA model to −10.1 kcal/mol in the extended monomer model (Figure 11A). Relative to BTMA, the DVB-BTMA^+^6:2 FTCA^−^ decomposition showed a larger positive Pauli term. The electrostatic, dispersion, and exchange-correlation contributions were more negative. For DVB-BTMA^+^PFHxA^−^, the final bond energy changed from −5.3 kcal/mol in BTMA to −8.7 kcal/mol in the extended monomer model (Figure 11B). The extended monomer decomposition also showed a larger positive Pauli term. The dispersion and exchange-correlation terms were more negative than in the BTMA energy decomposition.

PFOA and PFOS showed the same pattern (Figure 11C,D). For DVB-BTMA^+^PFOA^−^, the final bond energy changed from −4.4 kcal/mol in BTMA to −10.9 kcal/mol in the extended monomer model. Larger positive Pauli terms were observed. Electrostatic, orbital, dispersion, and exchange-correlation contributions were more negative for DVB-BTMA^+^PFOA^−^ than for BTMA^+^PFOA^−^. For DVB-BTMA^+^PFOS^−^, the final bond energy shifted from −3.7 kcal/mol in BTMA to −10.8 kcal/mol. Relative to BTMA, the DVB-BTMA^+^PFOS^−^ decomposition showed a larger Pauli term. The solvation term had a smaller contribution. The electrostatic term was also less negative. However, the orbital, dispersion, and exchange-correlation terms were all more negative than in the BTMA complex.

**Figure 12:**
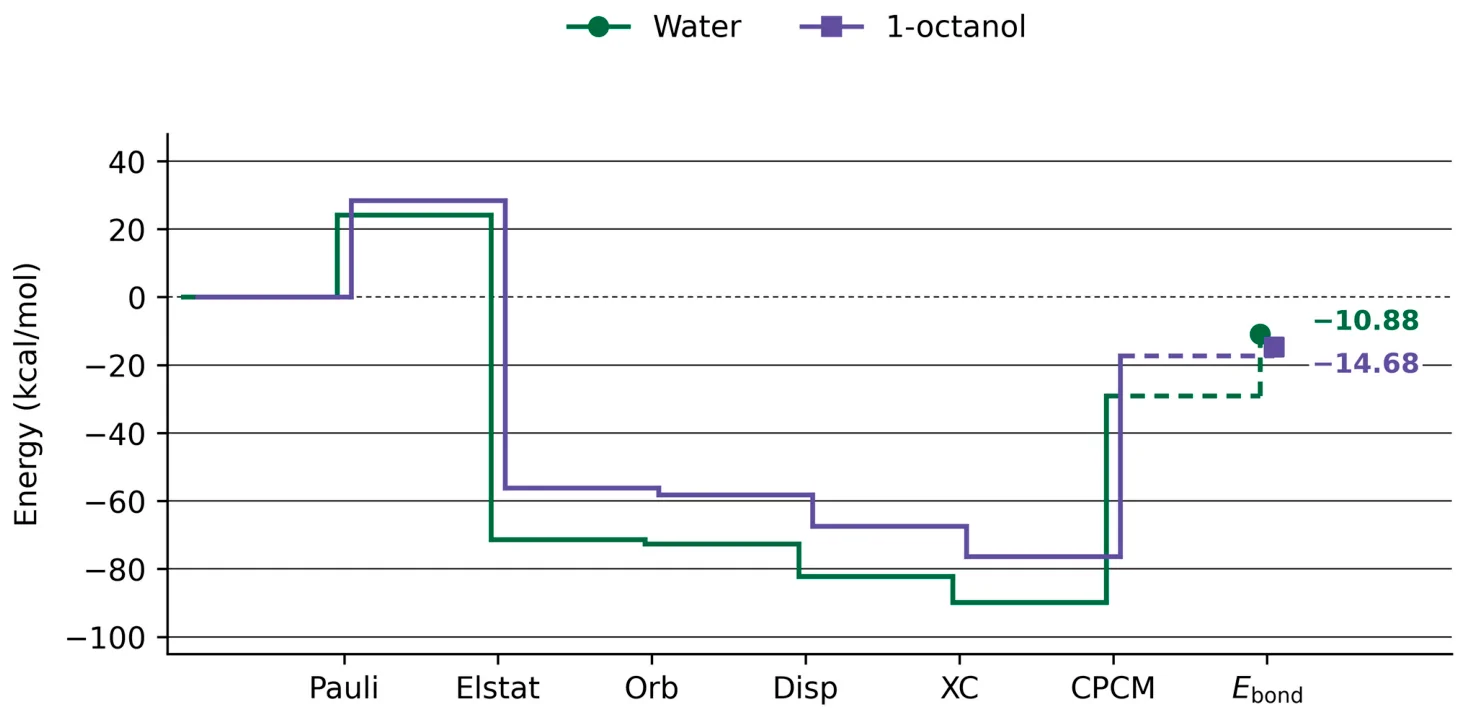
Energy-decomposition analysis of DVB-BTMA^+^PFOA^−^ in water and 1-octanol at ωB97X-D3/def2-TZVPD. Solid steps show the listed components. Dashed segments connect their sum to the ORCA Bond Energy. Values are in kcal/mol (Table 2 and Table S2).

For DVB-BTMA^+^PFOA^−^, the ORCA Bond Energy was −10.88 kcal/mol in water and −14.68 kcal/mol in 1-octanol. The orbital, exchange-correlation, and CPCM terms became more favorable in 1-octanol, while electrostatics, Pauli repulsion, and dispersion became less favorable. The component sum changed from −29.08 to −17.31 kcal/mol, despite the more negative endpoint. Bond Energy minus that sum was +18.20 kcal/mol in water and +2.62 kcal/mol in 1-octanol (Figure 12, Table 2 and Table S2).

### 3.3. Constrained Potential Energy Surface Scan of Local PFOA and Chloride Arrangements

**Figure 13:**
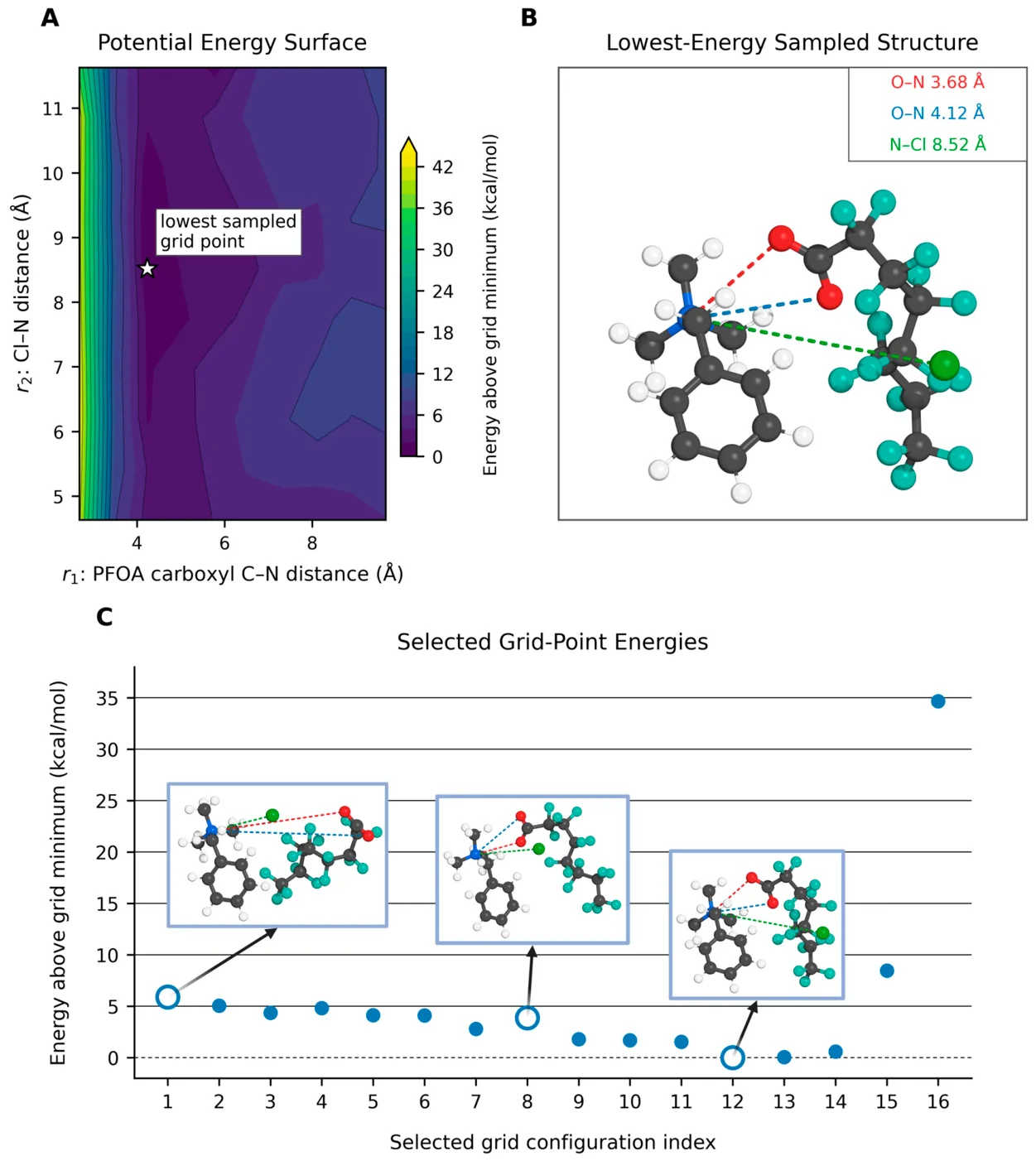
Constrained xTB/ALPB(water) scan of local PFOA, chloride, and BTMA arrangements. (**A**) Relative energies over 100 points constrained by PFOA carboxyl C–N (*r*_1_) and Cl–N (*r*_2_) distances. (**B**) Lowest-energy sampled structure with two O–N separations of 3.68 and 4.12 Å and Cl–N separation of 8.52 Å. (**C**) Selected grid-point energies and molecular geometries. All displayed energies are relative to the lowest sampled point and are in kcal/mol. The lowest sampled point is taken to have an energy above the grid minimum of 0.0 kcal/mol. Created in BioRender. Zwa, R. (2026) https://BioRender.com/j0xmdh7 (accessed on 1 October 2026).

The constrained two-dimensional PES sampled local arrangements of PFOA, chloride, and BTMA across the two intermolecular distance coordinates. The full grid spanned a relative electronic energy range of approximately 0–43.4 kcal/mol. In the lowest-energy grid structure, the two PFOA carboxylate oxygen to ammonium nitrogen distances were 4.1 Å and 3.7 Å, while the chloride to nitrogen distance was 8.5 Å. This structure shows one low-energy constrained arrangement with PFOA near the ammonium site and chloride farther from direct coordination (Figure 13A,B).

Selected grid configurations include chloride near the ammonium group with more distant PFOA, configurations with both anions near the ammonium group, and configurations with PFOA near the ammonium group while chloride is farther away. The high-energy terminal point reflects the imposed distance constraints at that grid location (Figure 13C). The relative energies for all 100 grid points are provided in the Supplementary Data (Table S5).

### 3.4. Molecular Dynamics of pVBTMA_12_ and PFOA

The simulated system and its structural and parameterization checks are documented (Table S6a,b). Each of the three replicas underwent separate equilibration before a 10 ns simulation (Table S7).

**Figure 14:**
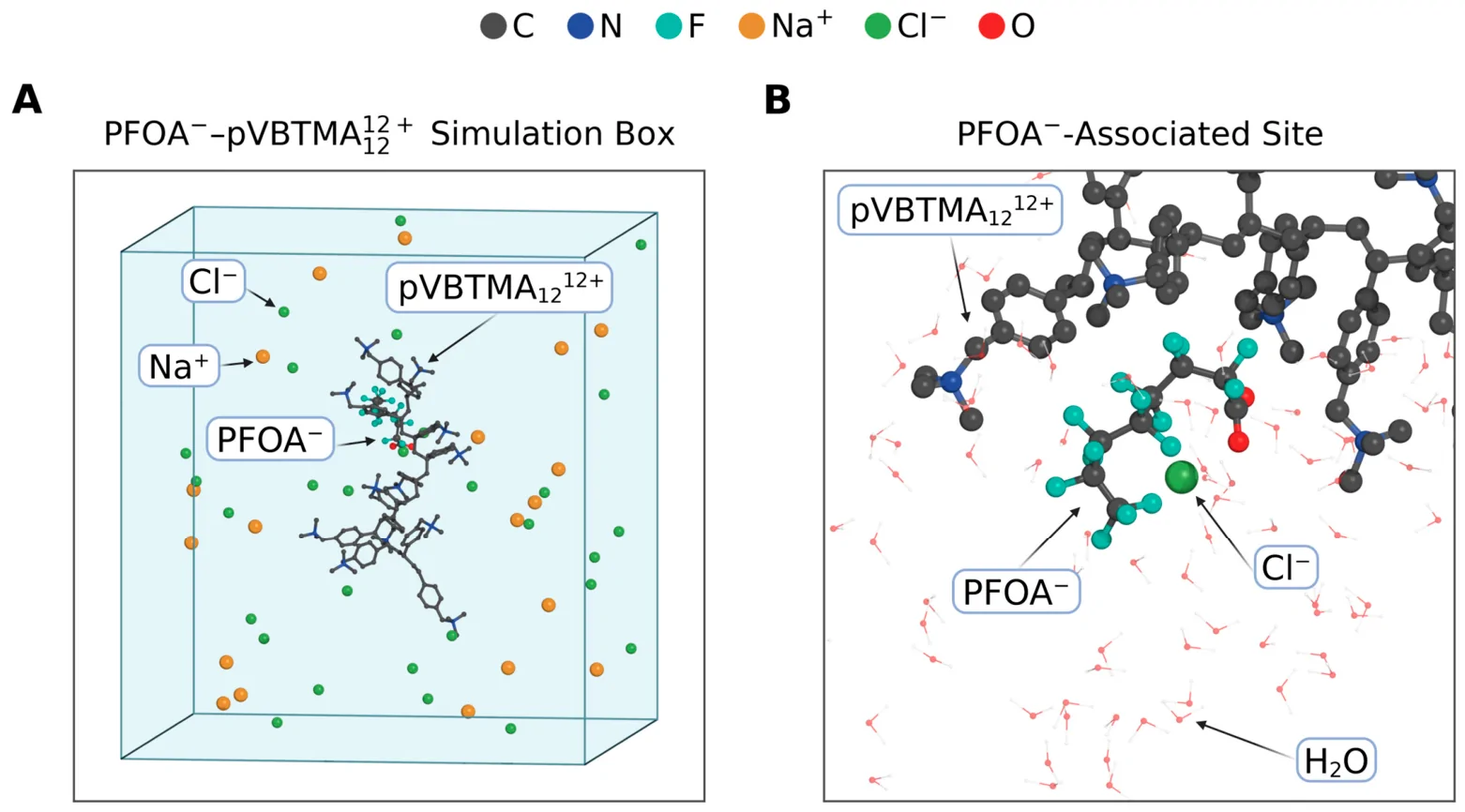
Observed PFOA-associated pVBTMA_12_^12+^ oligomer configuration from replica 1 at 9.856 ns. (**A**) Periodic cell containing the oligomer, PFOA^−^, 19 Na^+^, and 30 Cl^−^, with water omitted for clarity. (**B**) Detail of the PFOA-associated site with a uniform sample of complete water molecules across the field of view. Local ions are displayed within 8 Å of PFOA. One chloride and no sodium satisfy this cutoff. The atomic identity legend colors correspond directly to atomic colors in the rendered structures. Hydrogen atoms in water molecules are rendered as white. Created in BioRender. Zwa, R. (2026) https://BioRender.com/z9bjbqz (accessed on 7 October 2026).

PFOA remained in close contact with the oligomer during all three 10 ns trajectories. Mean minimum PFOA-to-oligomer heavy-atom distances were 3.006, 3.014, and 2.974 Å in replicas 1, 2, and 3, respectively. A frame from replica 1 at 9.856 ns shows the hydrated local environment (Figure 14A,B). Raw RMSD, radius of gyration, headgroup distance, tail-contact, and hydration traces accompany the sampling summaries (Figure S1A–E, Tables S8–S10).

**Figure 15:**
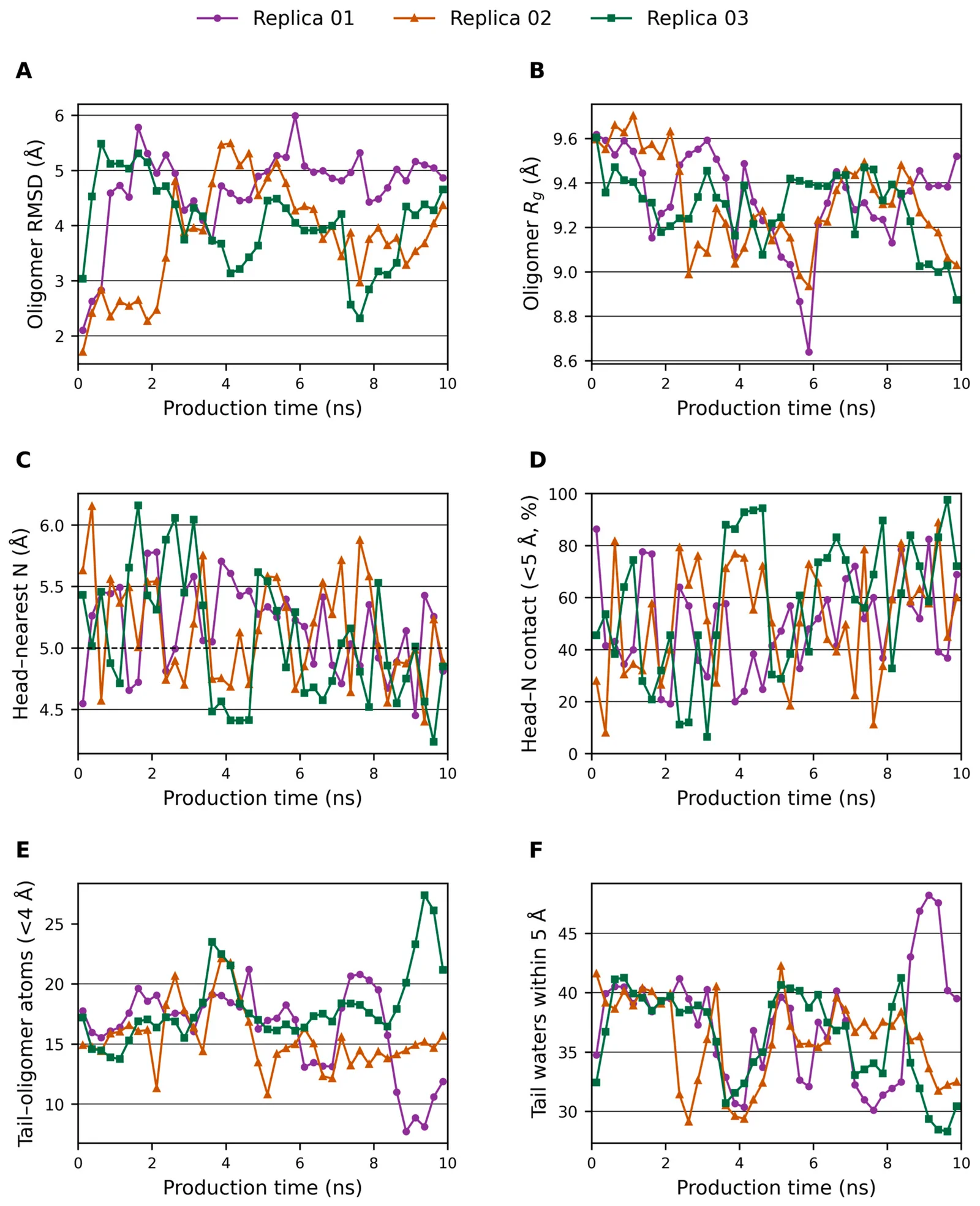
Temporal averages for three 10 ns molecular dynamics replicas. (**A**) Oligomer heavy-atom RMSD, (**B**) mass-weighted radius of gyration, (**C**) nearest PFOA headgroup O–ammonium N distance, (**D**) percentage of frames with an O–N separation below 5 Å, (**E**) unique oligomer heavy atoms within 4 Å of the fluorinated PFOA tail, and (**F**) waters within 5 Å of that tail. Symbols show means over consecutive, nonoverlapping 0.25 ns windows containing 125 frames. Replica 1 uses purple circles, replica 2 orange triangles, and replica 3 green squares.

The oligomer continued to rearrange during production. Mean radii of gyration were 9.335, 9.312, and 9.293 Å in the three replicas. Estimated RMSD integrated autocorrelation times were 0.344, 0.951, and 0.524 ns, with effective sample sizes of 14.5, 5.3, and 9.5. These correlation times exceeded the 0.25 ns display window, so the displayed means were not treated as independent samples. All three RMSD effective sample sizes were below 20. The first-to-last standardized shifts in radius of gyration were 0.61, 1.85, and 2.21, outside the 0.5 screen. Comparable replica means therefore did not establish global structural convergence within 10 ns (Figure 15A,B, Tables S8–S10).

The mean nearest carboxylate oxygen to any ammonium nitrogen distances were 5.165, 5.148, and 5.055 Å in the three replicas. The contact occupancies for separations below 5 Å were 49.7%, 51.6%, and 57.9%. The supplementary 1 ns contact blocks ranged from 28.6% to 77.8%. The fluorinated tail contacted means of 16.27, 15.49, and 18.01 oligomer heavy atoms, while waters within 5 Å averaged 37.41, 36.06, and 36.23. PFOA maintained close contact with the oligomer, while headgroup and tail environments varied with time and replica. These data do not identify a fixed ammonium site or equilibrium occupancy (Figure 15C–F, Tables S8–S11).

## 4. Discussion

### 4.1. BTMA Model Exchange Energetics and Interaction Analysis

#### 4.1.1. Electronic Energies of Exchange Vary Between r^2^SCAN-3c and ωB97X-D3

In BTMA, exchange compares PFAS and chloride at one cationic site. The small electronic exchange energies reflect a close balance between these ion pairs and their aqueous anion references. PFHxA gave the most favorable electronic exchange at r^2^SCAN-3c, while 6:2 FTCA gave the most favorable value at ωB97X-D3. This change in ordering shows that the selected geometries and functional influence the relative energies, alongside PFAS headgroup and chain structure (Figure 4A).

A common isolated-anion reference makes the BTMA and extended-model comparisons directly comparable at each functional. The BTMA electronic energies span only a few kcal/mol, so their ordering should be interpreted in the context of the selected molecular states. Sampling of alternative BTMA complex geometries was outside the conformer test reported here (Figure 4A).

#### 4.1.2. Thermochemical Corrections Give Positive BTMA Exchange Free Energies

Both methods support that PFAS anion exchange at an isolated BTMA site is thermodynamically unfavorable in the selected states for all four PFAS (Figure 4B).

Positive BTMA exchange free energies show that local ammonium to PFAS headgroup interaction alone was insufficient to favor exchange with chloride in these cycles. The positive net thermochemical corrections outweighed the small electronic contributions. Backbone contacts and local solvation can alter this balance in larger models, alongside the geometry and thermochemistry of the bound species (Figure 4B and Figure 5A–C).

#### 4.1.3. Functional Dependence Reflects Electronic and Thermochemical Contributions

The net thermochemical correction was positive for both functionals but smaller at ωB97X-D3 than at r^2^SCAN-3c. Its effect on the functional comparison depended on the electronic difference. Both contributions favored ωB97X-D3 for 6:2 FTCA. For PFOA, its less favorable hybrid electronic exchange outweighed the smaller correction, leaving a higher Gibbs exchange energy. The two effects nearly canceled for PFHxA (Figure 5A–C and Figure 6A,B).

Several species contribute to the functional difference in Δ(*G* − *E*_el_)_exchange_. The BTMA^+^P^−^ complex terms increased the ωB97X-D3 correction relative to r^2^SCAN-3c, while the BTMA^+^Cl^−^ reference and isolated PFAS anion terms decreased the correction (Figure 6A). The free chloride term was negligible at the displayed precision. Analyzing Δ(*G* − *E*_el_)_exchange_ components (zero-point energy, thermal corrections, and entropy) shows that the entropy term accounted for most of the difference in Δ(*G* − *E*_el_)_exchange_. Zero-point energy and finite-temperature thermal correction terms were small for all four PFAS (Figure 6B).

Quasi-RRHO treatment regularizes the entropy from very low vibrational frequencies. These modes can include movement of the PFAS headgroup near ammonium, methyl rotation, and fluorinated-chain torsions. Different optimized geometries can therefore change the thermochemical correction. Quasi-RRHO treatment addresses low-frequency vibrational entropy, while populations of multiple molecular conformations contribute separately to free energy.

#### 4.1.4. Energy Decomposition Shows That BTMA^+^P^−^ Interaction Is Mainly Due to Electrostatics

Electrostatic attraction was the largest stabilizing term in every complex. This ordering indicates a local ion-pairing mechanism at the BTMA site, where the anionic PFAS headgroup is drawn toward the quaternary ammonium group. The positive solvation terms show that continuum solvent treatment offsets part of the gas-phase electrostatic stabilization. As a result, the final bond energies were less negative than the electrostatic term (Figure 7A–D).

PFHxA gave the most favorable final bond energy with both functionals, followed by PFOA and PFOS. This ordering indicates that the carboxylate PFAS were at least as favorable as the sulfonate PFAS in the isolated BTMA binding model. The comparison between PFHxA and PFOA also shows that the shared carboxylate headgroup does not determine differences in EDA components by itself. Chain length may change the balance among electrostatics, Pauli repulsion, and dispersion. 6:2 FTCA, however, was slightly unfavorable with r^2^SCAN-3c, but favorable with ωB97X-D3. 6:2 FTCA may be more sensitive to functional choice than the other PFAS (Figure 7A–D).

The selected BTMA ion pairs generally had negative ORCA Bond Energy, except for the 6:2 FTCA structure at r^2^SCAN-3c. Stable local complexes may therefore form at one ammonium site even when the full anion exchange free energy remains unfavorable.

### 4.2. Extended Monomer Exchange Energetics and Interaction Analysis

#### 4.2.1. Extended Monomer Electronic Energies Favor Additional Resin Contacts

The comparison between BTMA and the extended monomer shows that model size changes the electronic exchange trend. All four electronic exchange energies became more negative on expanding BTMA to DVB-BTMA^+^. The changes were −3.67, −5.03, −10.37, and −8.66 kcal/mol for 6:2 FTCA, PFHxA, PFOA, and PFOS, respectively. This shift indicates that the additional backbone area stabilizes PFAS exchange at the electronic level. Both headgroup classes showed this effect (Figure 9A). The BTMA model primarily tests the anionic headgroup at one ammonium site. The extended monomer also models interactions between PFAS tails and the resin backbone. This added interaction favored the longer PFOA and PFOS structures more than PFHxA or 6:2 FTCA (Figure 9A).

#### 4.2.2. Extended Monomer Free Energies Show That Model Size Changes PFAS Ranking

With matched free-ion references, expansion lowered Gibbs exchange energies appreciably for PFOA, PFOS, and PFHxA. The change for 6:2 FTCA was only −0.17 kcal/mol, although its electronic exchange energy decreased by −3.67 kcal/mol. Its thermochemical correction therefore offset most of the electronic model-size effect. Added resin contacts produced different net free energy changes for the four selected PFAS (Figure 9A,B).

The larger model-size effect for PFOA than PFHxA is consistent with contributions from contacts along the longer fluorinated chain. However, conformer selection also affects these comparisons. The r^2^SCAN-3c PFOA substitutions span −1.71 to +0.80 kcal/mol, while its original r^2^SCAN-3c and ωB97X-D3 cycles lie on opposite sides of zero. This supports treating near-zero signs and close PFAS rankings as sensitive to the chosen molecular states. The r^2^SCAN-3c substitution span describes sensitivity within the tested states and does not quantify uncertainty in the hybrid results (Figure 9B, Table S4a).

Experimental findings provide a limited comparison with these molecular trends. Poblete et al. observed PFOS sequestration by cholestyramine after 48 h under in vitro ruminal conditions [13]. This supports PFOS association with the resin in a biological medium, but does not test the present chloride exchange energies or the four-compound ranking. Ahmadi et al. studied a different quaternary ammonium styrene-divinylbenzene resin in synthetic groundwater [14]. At low concentrations, PFOS had greater adsorption affinity than PFOA, and PFOA had greater affinity than PFHxA. Maximum adsorption capacities followed the opposite order for these three compounds. PFOS also suppressed PFOA uptake in simultaneous binary mixtures. The PFOA/PFHxA affinity trend is qualitatively consistent with the extended monomer results, whereas the PFOS/PFOA affinity trend differs from the present aqueous exchange ordering. Adsorption capacity and competitive uptake therefore cannot be inferred directly from the molecular exchange ranking. The authors interpreted their data using differences in site accessibility and pore blocking. The reduced DFT models omit these effects [14].

#### 4.2.3. Similar Bound-State Solvent Shifts Leave Little Change in Exchange

The PFOA exchange cycle changed little when the bound-state continuum was changed from water to 1-octanol. The PFOA complex Gibbs energy decreased by 8.60 kcal/mol, while the chloride complex decreased by 8.86 kcal/mol. These shifts nearly canceled in the exchange cycle, leaving a +0.26 kcal/mol Gibbs change. Electronic exchange changed by only +0.20 kcal/mol. Thus, the two continuum treatments gave similar net exchange energies despite larger changes in the individual bound states (Figure 10A,B, Table S3a–c).

The isolated anions remain aqueous in both cycles, so this comparison tests solvent treatment of the resin-bound species. Reaction-field calculations on stacked cytosine dimers likewise illustrate that continuum solvent effects depend on the molecular states being compared [57]. For the present PFOA cycle, the small solvent difference and near-zero Gibbs values provide no general solvent-affinity trend for PFAS uptake.

#### 4.2.4. Energy-Decomposition Analysis Shows How the Extended Monomer Stabilizes PFAS Binding

The extended monomer EDA shows that the larger cholestyramine model changes both favorable and unfavorable energy terms. Electrostatic attraction remained the largest stabilizing contribution for all four PFAS, which is consistent with ion pairing between the anionic headgroup and the ammonium site. Model expansion also increased Pauli repulsion for every PFAS. This reflects more close contacts in the selected extended monomer structures. Even with these added penalties, the final bond energies became more negative for all four PFAS in the extended monomer model (Figure 11A–D).

The added stabilization did not arise from a single EDA term for any of the four PFAS. For 6:2 FTCA and PFOA, the extended monomer made the electrostatic term more negative and also increased dispersion and exchange-correlation stabilization. For DVB-BTMA^+^PFHxA^−^ and DVB-BTMA^+^PFOS^−^, electrostatic attraction became less negative in the extended monomer model. Their reported Bond Energies nevertheless became more negative, alongside changes in the other printed terms. PFHxA gained stronger dispersion and exchange-correlation stabilization, while the reported continuum contribution changed. PFOS followed the same pattern more strongly, with a change in the reported continuum term and larger dispersion and exchange-correlation contributions in the extended model (Figure 11A–D). EDA describes selected structures relative to frozen fragments. Dispersion is one interaction component, while a solvent-driven hydrophobic free energy would also require changes in solvent organization.

The strongest final stabilization in the extended monomer model occurred for PFOA and PFOS. Their final bond energies were nearly identical and were more negative than those of 6:2 FTCA and PFHxA. The model-size changes in Gibbs exchange were also largest for PFOA and PFOS. The near-identical EDA endpoints, however, provide a comparison of selected ion pairs rather than a resolved selectivity ordering. The distinct behavior of PFHxA and PFOA is especially useful for comparison because both contain carboxylate headgroups. The change in relative favorability between these two PFAS is consistent with a contribution from backbone contacts to exchange in the modeled complexes.

For PFOA, the more negative 1-octanol Bond Energy contrasts with the slightly less favorable exchange free energy. EDA compares one ion pair with frozen fragments. The exchange cycle additionally includes the optimized chloride pair, aqueous free ions, and thermochemical corrections. The solvent changes in the two bound complexes largely cancel in exchange, whereas the printed EDA component sum and Bond Energy respond differently to the solvent treatment (Figure 10A,B and Figure 12, Table S2).

#### 4.2.5. NBO Analysis Shows Weak Donor-Acceptor Interactions in the DVB-BTMA^+^PFOA^−^ Complex

NBO analysis identified donation from the PFOA O72 lone pair into a nearby C13-H38 antibonding orbital of DVB-BTMA^+^ (Figure 8). The *E*^(2)^ stabilization energy for this interaction was only 0.20 kcal/mol, indicating that it provides a small contribution to stabilization. This is consistent with the results of EDA, where orbital interactions were stabilizing but much smaller than the electrostatic contribution.

### 4.3. The Constrained PES Samples One Possible Local Exchange Arrangement

The constrained PES contains relatively low-energy arrangements with PFOA near the BTMA ammonium group and chloride farther from direct coordination. It describes the relative energies of constrained local structures. The grid supplies neither a transition state nor an activation barrier, and the ordering of sampled points cannot establish a minimum free-energy pathway. The low-energy geometry is therefore one possible local arrangement relevant to exchange (Figure 13A–C).

### 4.4. PFOA Contact Persists During Oligomer Rearrangement

The PFOA-to-oligomer minimum distance remained close to 3 Å in all replicas, supporting persistent local contact over the sampled 10 ns interval. Replica 1 still showed an early-to-late standardized shift of 0.78, above the 0.5 screen. Its corresponding distances changed from 2.975 to 3.087 Å. Local contact persistence and adequate sampling of a mean are therefore separate observations (Tables S8 and S10).

Ammonium contact was more variable. The overall contact occupancies for separations below 5 Å were similar across replicas, but the 1 ns block values varied widely. PFOA remained polymer-associated while its carboxylate headgroup sampled different local relationships with the ammonium sites. Tail contacts and hydration changed alongside the headgroup environment (Figure 14B and Figure 15C–F).

The slow RMSD and shape observables had low effective sample sizes or measurable drift, despite similar replica means. Global structural convergence was therefore not established within 10 ns. The 0.25 ns averages show temporal behavior at a common resolution across the six observables, while the full-resolution correlations and original 1 ns summaries provide the sampling diagnostics. Longer trajectories would be needed to improve estimates for these slow oligomer motions (Figure 15A,B, Tables S8–S10).

The PES and MD calculations describe different aspects of the molecular model. The PES samples local configurations of two anions and one BTMA site. MD follows a hydrated PFOA-associated oligomer state prepared before production, with a distant chloride removed to preserve neutrality. It shows contact persistence and rearrangement of that state. Determining a chloride-displacement pathway or an exchange free energy would require sampling transitions between defined states (Figure 13A–C and Figure 14A,B).

## 5. Rigor and Reproducibility

Optimized DFT structures used for thermochemical analysis were checked for imaginary frequencies. DefGrid3 integration and TightOpt convergence criteria were used for the quantum chemical calculations. The pVBTMA_12_ structural, charge-transfer, and topology checks are summarized with their criteria and recorded outcomes (Table S6b). The periodic cell series passed the trajectory checks described in Section 2.6.4. Nine r^2^SCAN-3c conformer sensitivity frequency outputs terminated normally with no imaginary modes (Table S4a). Deposited data and scripts are described in the Data and Code Availability section. Representative computational requirements for the major calculations are summarized (Table 3).

### Data and Code Availability

All calculation files, source structures, plotting data, analysis scripts, Supplementary Tables, and workflow code associated with this study are archived in Zenodo as release v1.0.0 at https://doi.org/10.5281/zenodo.23087140 (accessed on 1 October 2026) and organized within the Supplementary_Materials directory.

Large text files that exceed upload limits are provided as compressed files. Full details for each calculation can be found in the output files in each calculation folder.

The file figure_data_and_code_map.md identifies the script and extracted data associated with each figure group, while calculation_folder_map.md identifies the calculation folders that contain the underlying input and output files. Source structures and model construction inputs are described in source_structures_map.md.

The Streamlit graphical user interface source code and documentation are included in the same repository, and the deployed web application is available at https://pfas-removal.streamlit.app/ (accessed on 1 October 2026). Additional intermediate files and large raw outputs that are not included in the supplementary archive can be provided upon request.

## 6. Strengths and Limitations

This study uses density functional theory to examine PFAS exchange with cholestyramine model systems through electronic energies, Gibbs free energies, thermochemical corrections, and interaction components. The comparison between the BTMA and extended monomer models, along with density functional comparisons within each model, allows analysis of the relative contributions of electrostatics and noncovalent interactions to anion exchange.

The reduced DFT structures represent one ammonium site and a larger resin fragment. A complete cholestyramine bead contains a heterogeneous crosslinked network with many interacting sites. Static thermochemistry treats individual optimized structures. Low-frequency regularization and the limited r^2^SCAN-3c PFOA conformer test only partly address the flexibility of these species. Additional final-level conformer sampling and conformational populations would be needed to assess the robustness of close hybrid exchange comparisons.

Continuum solvents approximate hydration around the ion pairs. Explicit waters can change relative interaction energies, as also found in cytosine-base models [58], and may alter the resin-bound geometries studied here. The finite linear pVBTMA_12_ model omits crosslinks and resin-bead heterogeneity. Its three trajectories share one initial associated pose, and several slow observables remain insufficiently sampled. Force-field approximations and limited exploration of other associated states also restrict extrapolation to the full hydrated resin.

Only chloride was modeled as a competing anion. Bile acids and other intestinal anions could change PFAS occupancy at resin sites. Albumin release, biological transport, resin capacity, and uptake kinetics would require separate calculations or experiments. The published adsorption comparisons concern different materials or conditions and provide qualitative context rather than direct validation of the calculated exchange energies. The biological interpretation remains a molecular hypothesis.

## 7. Future Directions

Molecular dynamics simulations of larger and crosslinked resin models could test how network structure changes hydration, ion accessibility, and slow polymer motion. Longer replicas would improve estimates for the slow structural observables identified here. Umbrella sampling or alchemical free energy methods would be required for quantitative binding or exchange free energies. Representative hydrated structures could also support later QM/MM calculations. Comparisons with human serum albumin would require studies of PFAS release and transfer under biological competition.

The EDA results suggest two controlled design comparisons. One would compare benzyl and biphenyl groups adjacent to an otherwise identical trimethylammonium site, holding the site count and formal charge fixed. This would test whether added aromatic contact area improves dispersion and total exchange free energy without excessive Pauli repulsion. A second comparison would vary the methylene spacer between the aromatic group and ammonium center while keeping one cationic site. This would test whether the PFAS headgroup can maintain electrostatic contact while the tail contacts the backbone. Both proposals follow the competing EDA contributions observed upon model expansion (Figure 11A–D). Neither design has been evaluated here. Improved selectivity would require subsequent testing under hydrated, competitive conditions.

## 8. Conclusions

This study examined PFAS exchange with cholestyramine models that separate local ammonium ion pairing from added resin contacts. BTMA exchange free energies were positive for all four PFAS. With common aqueous free-anion references, the extended monomer lowered electronic exchange energies for every compound, while the Gibbs improvement varied from negligible for 6:2 FTCA to appreciable for PFOA. Similar solvent shifts in the PFOA- and chloride-bound complexes left little net change between water and 1-octanol.

Electrostatic attraction was the largest stabilizing EDA component in both resin models. Added backbone contacts changed dispersion and exchange-correlation terms alongside Pauli repulsion. These structural contributions can favor exchange, although near-zero free energies remain sensitive to the selected molecular states. The hydrated oligomer trajectories showed persistent PFOA contact with changing headgroup and tail environments, consistent with association across multiple local arrangements.

The combined results provide molecular hypotheses for testing how resin contact area and ammonium geometry affect PFAS exchange. The constrained PES and associated-state simulations supply structural context for these comparisons. Experimental exchange measurements under controlled hydration and biological competition would test their relevance to cholestyramine sequestration.

## Figures and Tables

**Table 1:** Principal DFT anion exchange energies. Values are given in kcal/mol for the exchange cycle defined in Equation (1). Gibbs free energies were evaluated at 310.15 K. The bound-state solvent applies to both the PFAS complex and the chloride reference complex. Isolated anions were modeled in water. The PFOA 1-octanol row uses SMD/CPCM 1-octanol with *ε* = 9.8629. The same aqueous free-anion reference is used for both resin models at each functional.

Model	Functional	Bound-State Solvent	PFAS	Δ*E*_exchange_	Δ*G*_exchange_
BTMA^+^	r^2^SCAN-3c	Water	6:2 FTCA	−1.07	5.99
BTMA^+^	r^2^SCAN-3c	Water	PFHxA	−2.69	4.52
BTMA^+^	r^2^SCAN-3c	Water	PFOA	−1.03	7.36
BTMA^+^	r^2^SCAN-3c	Water	PFOS	−1.04	6.26
BTMA^+^	ωB97X-D3	Water	6:2 FTCA	−3.13	2.07
BTMA^+^	ωB97X-D3	Water	PFHxA	−2.04	4.53
BTMA^+^	ωB97X-D3	Water	PFOA	0.82	8.07
BTMA^+^	ωB97X-D3	Water	PFOS	−0.46	5.73
DVB-BTMA^+^	ωB97X-D3	Water	6:2 FTCA	−6.80	1.90
DVB-BTMA^+^	ωB97X-D3	Water	PFHxA	−7.07	1.68
DVB-BTMA^+^	ωB97X-D3	Water	PFOA	−9.54	−0.45
DVB-BTMA^+^	ωB97X-D3	Water	PFOS	−9.12	1.04
DVB-BTMA^+^	ωB97X-D3	1-octanol	PFOA	−9.34	−0.20

**Table 2:** EDA components and ORCA Bond Energy for selected PFAS ion pairs. (**a**) BTMA^+^ functional comparison, (**b**) DVB-BTMA^+^ in water, and (**c**) the PFOA solvent comparison. Values are in kcal/mol. Hybrid calculations use ωB97X-D3/def2-TZVPD. Solvation uses CPCM/SMD. XC is the printed Δ*E*^0^(XC) term, and CPCM is the dielectric contribution. A dash denotes gCP not separately reported. Bond Energy uses the frozen fragments at the complex geometry. The aqueous PFOA row in (**c**) repeats the value in (**b**).

(a) BTMA^+^ ion pairs in water									
**PFAS**	**Method or Solvent**	**Pauli**	**Electrostatic**	**Orbital**	**Dispersion**	**Δ*E*^0^(XC)**	**gCP**	**CPCM**	**Bond Energy**
6:2 FTCA	r^2^SCAN-3c	15.73	−97.51	−0.90	−0.97	−5.06	0.11	78.15	5.71
6:2 FTCA	ωB97X-D3	10.34	−102.64	−0.70	−1.84	−3.56	−	79.40	−2.92
PFHxA	r^2^SCAN-3c	8.48	−88.63	−1.00	−1.70	−6.64	0.28	70.54	−5.01
PFHxA	ωB97X-D3	12.93	−94.36	−0.87	−3.56	−4.16	−	71.16	−5.28
PFOA	r^2^SCAN-3c	6.30	−88.15	−0.58	−1.47	−5.15	0.19	68.76	−4.11
PFOA	ωB97X-D3	9.26	−92.24	−0.50	−2.99	−3.10	−	69.27	−4.39
PFOS	r^2^SCAN-3c	5.49	−86.47	−0.57	−1.41	−4.44	0.12	68.48	−3.32
PFOS	ωB97X-D3	7.82	−89.25	−0.44	−2.73	−2.72	−	68.34	−3.66
(b) DVB-BTMA^+^ ion pairs in water at ωB97X-D3									
**PFAS**	**Method or Solvent**	**Pauli**	**Electrostatic**	**Orbital**	**Dispersion**	**Δ*E*^0^(XC)**	**gCP**	**CPCM**	**Bond Energy**
6:2 FTCA	water	25.77	−114.84	−1.59	−8.61	−8.38	−	80.23	−10.10
PFHxA	water	21.18	−92.91	−1.19	−7.56	−6.70	−	61.92	−8.71
PFOA	water	24.16	−95.53	−1.25	−9.55	−7.71	−	60.80	−10.88
PFOS	water	27.54	−68.60	−1.35	−10.79	−9.13	−	34.28	−10.81
(c) DVB-BTMA^+^PFOA^−^ in water and 1-octanol at ωB97X-D3									
**PFAS**	**Method or Solvent**	**Pauli**	**Electrostatic**	**Orbital**	**Dispersion**	**Δ*E*^0^(XC)**	**gCP**	**CPCM**	**Bond Energy**
PFOA	water	24.16	−95.53	−1.25	−9.55	−7.71	−	60.80	−10.88
PFOA	1-octanol	28.40	−84.52	−2.08	−9.23	−8.92	−	59.04	−14.68

**Table 3:** Representative computational requirements. Wall times are medians and observed ranges from terminal ORCA or NAMD timing records. Quantum rows use parent-job runtimes, without adding separately reported EDA fragment runtimes. The MD row sums ten 1 ns production segments per replica and excludes preparation and equilibration. ORCA MaxCore is a configured per-process limit, not measured peak memory.

Calculation	Model	Runs (*n*)	Compute Setup	Wall Time, Median (Range)
r^2^SCAN-3c optimization + frequency	BTMA^+^P^−^	4	16 cores; 8.0 GB MaxCore/process	2 h 9 min (1 h 34 min to 3 h 14 min)
ωB97X-D3 optimization	BTMA^+^P^−^	4	64 cores; 2.5 GB MaxCore/process	1 h 59 min (1 h 18 min to 2 h 22 min)
ωB97X-D3 frequency	BTMA^+^P^−^	4	32 cores; 8.5 GB MaxCore/process	2 h 8 min (1 h 49 min to 3 h 58 min)
ωB97X-D3 EDA-NOCV	BTMA^+^P^−^	4	32 cores; 4.0 GB MaxCore/process	15 min 23 s (12 min 36 s to 19 min 7 s)
GFN2-xTB GOAT conformer search	DVB-BTMA^+^P^−^	4	64 cores; 2.5 GB MaxCore/process	4 h 32 min (3 h 43 min to 7 h 54 min)
r^2^SCAN-3c optimization	DVB-BTMA^+^P^−^	4	64 cores; 2.5 GB MaxCore/process	10 h 44 min (2 h 47 min to 18 h 58 min)
ωB97X-D3 optimization	DVB-BTMA^+^P^−^	4	64 cores; 2.5 GB MaxCore/process	6 h 5 min (3 h 2 min to 7 h 41 min)
ωB97X-D3 frequency	DVB-BTMA^+^P^−^	4	32–64 cores; 8.5 GB MaxCore/process	5 h 53 min (3 h 57 min to 13 h 1 min)
ωB97X-D3 EDA-NOCV	DVB-BTMA^+^P^−^	4	32 cores; 4.0 GB MaxCore/process	46 min 17 s (39 min 13 s to 55 min 19 s)
ωB97X-D3 NBO 7.0.9	DVB-BTMA^+^PFOA^−^	1	64 cores; 6.0 GB MaxCore/process	42 min 15 s
Molecular dynamics, 10 ns production per replica	pVBTMA_12_/PFOA	3	8 CPU threads + NVIDIA GeForce RTX 4050 Laptop GPU	2 h 46 min (2 h 39 min to 2 h 49 min)

## Data Availability

The data generated in this study are presented in the main manuscript and Supplementary Materials. Additional computational data and workflow materials are archived in Zenodo as release v1.0.0 at https://doi.org/10.5281/zenodo.23087140 (accessed on 1 October 2026). The repository contains the main ORCA, PES, NBO, and molecular dynamics input and output files, source structures, model construction files, extracted plotting data, analysis and plotting scripts, Supplementary Tables, and workflow code used in this study. The repository also contains the materials used to reproduce the figures and molecular dynamics analyses described in the manuscript. The web-based ORCA input generation and output parsing interface is available at https://pfas-removal.streamlit.app/ (accessed on 1 October 2026). Additional intermediate files and large raw outputs that are not included in the repository can be provided upon request.

## Supplementary Materials

The following supporting information can be downloaded at: https://www.mdpi.com/article/10.3390/toxics14100901/s1, Figure S1: Raw molecular dynamics traces from the prepared pVBTMA_12_^12+^–PFOA^−^ system. Three 10 ns production replicas are shown at 2 ps sampling intervals. (A) Oligomer root mean square deviation (RMSD). (B) Radius of gyration. (C) Nearest PFOA carboxylate oxygen to any oligomer ammonium nitrogen. (D) Oligomer heavy atoms within 4 Å of the fluorinated tail. (E) Waters within 5 Å of the tail. The dashed line in panel C marks 5 Å. Production began from a prepared PFOA-associated configuration; Table S1: BTMA^+^ exchange energetics and source energies. (a) Electronic source energies and (b) Gibbs source energies are in Eh. (c) Exchange electronic energies and free energies are given in Eh and kcal/mol for the chloride/PFAS exchange cycle. A common aqueous free-anion reference is used for each PFAS and functional across the resin-model comparisons; Table S2: EDA components and frozen-fragment Bond Energy for selected BTMA^+^ and DVB-BTMA^+^ PFAS ion pairs. (a) Printed components and Bond Energy. (b) Their component sum and arithmetic Bond Energy minus sum. Values are in kcal/mol. The gCP dash denotes a term not separately reported in the hybrid outputs. All rows use CPCM/SMD water except the labeled 1-octanol row. DVB-BTMA^+^ rows use ωB97X-D3/def2-TZVPD; Table S3: BTMA^+^ and extended monomer exchange energetics in water and 1-octanol. DVB-BTMA^+^ denotes the extended styrene–divinylbenzene monomer. (a) Electronic source energies and (b) Gibbs source energies are in Eh. (c) Exchange electronic energies and free energies are in Eh and kcal/mol. The 1-octanol row reports PFOA. The same aqueous isolated-anion references are used in both models and both solvent cycles; Table S4: Aqueous r^2^SCAN-3c conformer sensitivity of the extended-monomer PFOA exchange cycle. (a) Source energies and one-species substitutions, with all other species fixed at the original candidates. Electronic and Gibbs energies are in Eh. Differences and exchange values are in kcal/mol. (b) Zero-based GOAT ensemble indices, search-level relative energies, and symmetry-aware heavy-atom RMSDs relative to the original candidate. RMSD uses rigid alignment with element- and connectivity-preserving atom permutations. The search energy reference is the original GOAT minimum for that species. Quasi-RRHO used a 100 cm^−1^ reference frequency and a separate 1 cm^−1^ cutoff; Table S5: Constrained PFOA carboxyl C–N and N–Cl two-distance potential energy surface. The 100 sampled geometries were optimized with xTB/ALPB(water) under the two listed distance constraints. Relative energies use the lowest sampled grid energy, −133.643493975080 Eh, as zero; Table S6a: Twelve-unit pVBTMA oligomer and solvated PFOA-associated system definition. The finite, hydrogen-capped oligomer has twelve quaternary ammonium sites and a formal charge of 12+. The complete simulation box is electrically neutral with the specified ions; Table S6b: Structural and parameterization checks for pVBTMA_12_. Results refer to the defined oligomer and the chloride-form solvated precursor, as indicated; Table S7: Molecular dynamics protocol and three production replicas. Each 10 ns production replica followed separate equilibration. Analysis sampled 5000 frames per replica at 2 ps intervals; Table S8: Per-replica molecular dynamics summaries. Each row summarizes 5000 sampled frames. IAT is integrated autocorrelation time. ESS is effective sample size. RMSD, radius of gyration (Rg), end-to-end, head–N, PFOA–oligomer, and periodic self-image metrics are distances in Å. Head–selected N uses the initially selected ammonium site. Head–any N uses the nearest of all twelve sites. Tail contacts and waters are counts. IAT includes half the zero-lag contribution and the initial positive normalized autocorrelation sequence. ESS is n divided by twice IAT in frames; Table S9: One-nanosecond block summaries from three 10 ns production replicas. Mean and SD are computed from sampled frames within each block. The ten metrics and their units follow Table S8. Blocks are indexed 1–10 separately for each replica; Table S10: Numerical sampling diagnostics for selected molecular dynamics metrics. Early and late means and the standardized shift summarize within-replica behavior. Replica mean range, pooled within-replica SD, and their ratio summarize between-replica spread; Table S11: PFOA headgroup contact fraction near ammonium by one-nanosecond block. The fraction is the proportion of sampled frames with a PFOA carboxylate oxygen at a distance of less than 5 Å from any oligomer ammonium nitrogen.
