# Supplementary material for: Anion Exchange Mechanism of PFAS with Cholestyramine: Implications for PFAS Removal from the Human Body
Source: Toxics. 2026 Oct 11;14(10):901. doi: 10.3390/toxics14100901 (PMC13709388; doi:10.3390/toxics14100901)
Supplement: Supplementary file 1 [file toxics-14-00901-s001.zip › toxics-4550545-supplementary.pdf]

Supplementary Materials

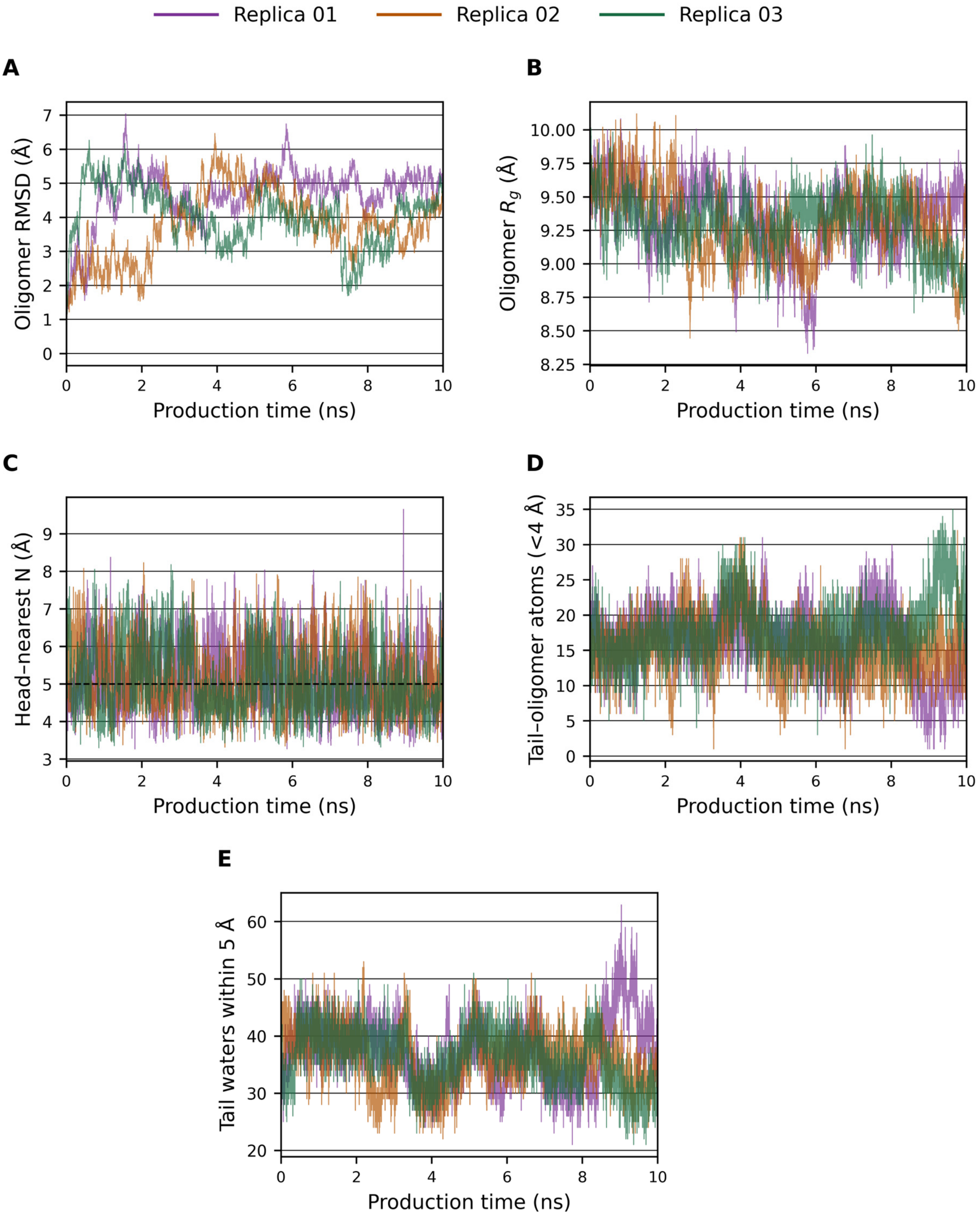

Figure S1. Raw molecular dynamics traces from the prepared pVBTMA$_{12}^{12+}$–PFOA$^-$ system. Three 10 ns production replicas are shown at 2 ps sampling intervals. (A) Oligomer root mean square deviation (RMSD). (B) Radius of gyration. (C) Nearest PFOA carboxylate oxygen to any oligomer ammonium nitrogen. (D) Oligomer heavy atoms within 4 Å of the fluorinated tail. (E) Waters within 5 Å of the tail. The dashed line in panel C marks 5 Å. Production began from a prepared PFOA-associated configuration.

(a)

Method	PFAS	E BTMA⁺P⁻ (Eh)	E BTMA⁺Cl⁻ (Eh)	E P⁻ (Eh)	E Cl⁻ (Eh)
r²SCAN-3c	6:2 FTCA	-2199.764389580	-905.517886846	-1754.590197254	-460.345405067
ωB97X-D3	6:2 FTCA	-2200.289787525	-905.731151435	-1754.938877499	-460.385228974
r²SCAN-3c	PFHxA	-1922.676121648	-905.517886846	-1477.499356306	-460.345405067
ωB97X-D3	PFHxA	-1923.137309076	-905.731151435	-1477.788129953	-460.385228974
r²SCAN-3c	PFOA	-2398.242098978	-905.517886846	-1953.067972856	-460.345405067
ωB97X-D3	PFOA	-2398.793370785	-905.731151435	-1953.448760993	-460.385228974
r²SCAN-3c	PFOS	-3071.263984706	-905.517886846	-2626.089844333	-460.345405067
ωB97X-D3	PFOS	-3071.948356352	-905.731151435	-2626.601702426	-460.385228974

(b)

Method	PFAS	G BTMA⁺P⁻ (Eh)	G BTMA⁺Cl⁻ (Eh)	G P⁻ (Eh)	G Cl⁻ (Eh)
r²SCAN-3c	6:2 FTCA	-2199.470781920	-905.315008170	-1754.526472190	-460.361149520
ωB97X-D3	6:2 FTCA	-2199.991676120	-905.524363850	-1754.871586160	-460.400973430
r²SCAN-3c	PFHxA	-1922.417866280	-905.315008170	-1477.471203550	-460.361149520
ωB97X-D3	PFHxA	-1922.874226070	-905.524363850	-1477.758058680	-460.400973430
r²SCAN-3c	PFOA	-2397.965154530	-905.315008170	-1953.023022170	-460.361149520
ωB97X-D3	PFOA	-2398.510894630	-905.524363850	-1953.400370720	-460.400973430
r²SCAN-3c	PFOS	-3070.979533810	-905.315008170	-2626.035645350	-460.361149520
ωB97X-D3	PFOS	-3071.656796230	-905.524363850	-2626.542534730	-460.400973430

(c)

Method	PFAS	ΔE (Eh)	ΔG (Eh)	ΔE (kcal/mol)	ΔG (kcal/mol)
r²SCAN-3c	6:2 FTCA	-0.001710547	0.009548920	-1.0734	5.9920
ωB97X-D3	6:2 FTCA	-0.004987564	0.003300460	-3.1297	2.0711
r²SCAN-3c	PFHxA	-0.004283562	0.007195920	-2.6880	4.5155
ωB97X-D3	PFHxA	-0.003256663	0.007223030	-2.0436	4.5325
r²SCAN-3c	PFOA	-0.001644343	0.011726290	-1.0318	7.3584
ωB97X-D3	PFOA	0.001312669	0.012866510	0.8237	8.0739
r²SCAN-3c	PFOS	-0.001658593	0.009970190	-1.0408	6.2564
ωB97X-D3	PFOS	-0.000731466	0.009128920	-0.4590	5.7285

Table S1. BTMA⁺ exchange energetics and source energies. (a) Electronic source energies and **(b)** Gibbs source energies are in Eh. **(c)** Exchange electronic energies and free energies are given in Eh and kcal/mol for the chloride/PFAS exchange cycle. A common aqueous free-anion reference is used for each PFAS and functional across the resin-model comparisons.

(a)

PFAS	Model and method	Pauli	Electrostatic	Orbital	Dispersion	ΔE⁰(XC)	gCP	CPCM	Bond Energy
6:2 FTCA	$BTMA^+$ (r^2SCAN-3c)	15.73	-97.51	-0.90	-0.97	-5.06	0.11	78.15	5.71
6:2 FTCA	$BTMA^+$ (ωB97X-D3)	10.34	-102.64	-0.70	-1.84	-3.56	–	79.40	-2.92
6:2 FTCA	DVB-$BTMA^+$ (water)	25.77	-114.84	-1.59	-8.61	-8.38	–	80.23	-10.10
PFHxA	$BTMA^+$ (r^2SCAN-3c)	8.48	-88.63	-1.00	-1.70	-6.64	0.28	70.54	-5.01
PFHxA	$BTMA^+$ (ωB97X-D3)	12.93	-94.36	-0.87	-3.56	-4.16	–	71.16	-5.28
PFHxA	DVB-$BTMA^+$ (water)	21.18	-92.91	-1.19	-7.56	-6.70	–	61.92	-8.71
PFOA	$BTMA^+$ (r^2SCAN-3c)	6.30	-88.15	-0.58	-1.47	-5.15	0.19	68.76	-4.11
PFOA	$BTMA^+$ (ωB97X-D3)	9.26	-92.24	-0.50	-2.99	-3.10	–	69.27	-4.39
PFOA	DVB-$BTMA^+$ (1-octanol)	28.40	-84.52	-2.08	-9.23	-8.92	–	59.04	-14.68
PFOA	DVB-$BTMA^+$ (water)	24.16	-95.53	-1.25	-9.55	-7.71	–	60.80	-10.88
PFOS	$BTMA^+$ (r^2SCAN-3c)	5.49	-86.47	-0.57	-1.41	-4.44	0.12	68.48	-3.32
PFOS	$BTMA^+$ (ωB97X-D3)	7.82	-89.25	-0.44	-2.73	-2.72	–	68.34	-3.66
PFOS	DVB-$BTMA^+$ (water)	27.54	-68.60	-1.35	-10.79	-9.13	–	34.28	-10.81

(b)

PFAS	Model and method	Component sum	Bond Energy	Bond minus sum
6:2 FTCA	$BTMA^+$ (r^2SCAN-3c)	-10.4488	5.7140	16.1628
6:2 FTCA	$BTMA^+$ (ωB97X-D3)	-19.0060	-2.9213	16.0847
6:2 FTCA	DVB-$BTMA^+$ (water)	-27.4231	-10.1024	17.3207
PFHxA	$BTMA^+$ (r^2SCAN-3c)	-18.6550	-5.0087	13.6463
PFHxA	$BTMA^+$ (ωB97X-D3)	-18.8623	-5.2796	13.5828
PFHxA	DVB-$BTMA^+$ (water)	-25.2649	-8.7084	16.5565
PFOA	$BTMA^+$ (r^2SCAN-3c)	-20.1026	-4.1147	15.9878
PFOA	$BTMA^+$ (ωB97X-D3)	-20.3077	-4.3912	15.9166
PFOA	DVB-$BTMA^+$ (1-octanol)	-17.3076	-14.6829	2.6247
PFOA	DVB-$BTMA^+$ (water)	-29.0785	-10.8754	18.2030
PFOS	$BTMA^+$ (r^2SCAN-3c)	-18.7883	-3.3234	15.4650
PFOS	$BTMA^+$ (ωB97X-D3)	-18.9931	-3.6645	15.3286
PFOS	DVB-$BTMA^+$ (water)	-28.0432	-10.8063	17.2368

Table S2. EDA components and frozen-fragment Bond Energy for selected $BTMA^+$ and DVB-$BTMA^+$ PFAS ion pairs. (a) Printed components and Bond Energy. **(b)** Their component sum and arithmetic Bond Energy minus sum. Values are in kcal/mol. The gCP dash denotes a term not separately reported in the hybrid outputs. All rows use CPCM/SMD water except the labeled 1-octanol row. DVB-$BTMA^+$ rows use ωB97X-D3/def2-TZVPD.

(a)

Model (solvent)	PFAS	E complex (Eh)	E chloride pair (Eh)	E P^- (Eh)	E Cl^- (Eh)
$BTMA^+$ / water	6:2 FTCA	-2200.289787525	-905.731151435	-1754.938877499	-460.385228974
$BTMA^+$ / water	PFHxA	-1923.137309076	-905.731151435	-1477.788129953	-460.385228974
$BTMA^+$ / water	PFOA	-2398.793370785	-905.731151435	-1953.448760993	-460.385228974
$BTMA^+$ / water	PFOS	-3071.948356352	-905.731151435	-2626.601702426	-460.385228974
DVB-$BTMA^+$ / 1-octanol	PFOA	-2865.805580031	-1372.727158116	-1953.448760993	-460.385228974
DVB-$BTMA^+$ / water	6:2 FTCA	-2667.277650980	-1372.713160371	-1754.938877499	-460.385228974
DVB-$BTMA^+$ / water	PFHxA	-2390.127333629	-1372.713160371	-1477.788129953	-460.385228974
DVB-$BTMA^+$ / water	PFOA	-2865.791899334	-1372.713160371	-1953.448760993	-460.385228974
DVB-$BTMA^+$ / water	PFOS	-3538.944164572	-1372.713160371	-2626.601702426	-460.385228974

(b)

Model (solvent)	PFAS	G complex (Eh)	G chloride pair (Eh)	G P^- (Eh)	G Cl^- (Eh)
$BTMA^+$ / water	6:2 FTCA	-2199.991676120	-905.524363850	-1754.871586160	-460.400973430
$BTMA^+$ / water	PFHxA	-1922.874226070	-905.524363850	-1477.758058680	-460.400973430
$BTMA^+$ / water	PFOA	-2398.510894630	-905.524363850	-1953.400370720	-460.400973430
$BTMA^+$ / water	PFOS	-3071.656796230	-905.524363850	-2626.542534730	-460.400973430
DVB-$BTMA^+$ / 1-octanol	PFOA	-2865.283177840	-1372.283465700	-1953.400370720	-460.400973430
DVB-$BTMA^+$ / water	6:2 FTCA	-2666.736924600	-1372.269344780	-1754.871586160	-460.400973430
DVB-$BTMA^+$ / water	PFHxA	-2389.623756470	-1372.269344780	-1477.758058680	-460.400973430
DVB-$BTMA^+$ / water	PFOA	-2865.269465380	-1372.269344780	-1953.400370720	-460.400973430
DVB-$BTMA^+$ / water	PFOS	-3538.409240800	-1372.269344780	-2626.542534730	-460.400973430

(c)

Model (solvent)	PFAS	ΔE (Eh)	ΔG (Eh)	ΔE (kcal/mol)	ΔG (kcal/mol)
$BTMA^+$ / water	6:2 FTCA	-0.004987564	0.003300460	-3.1297	2.0711
$BTMA^+$ / water	PFHxA	-0.003256663	0.007223030	-2.0436	4.5325
$BTMA^+$ / water	PFOA	0.001312669	0.012866510	0.8237	8.0739
$BTMA^+$ / water	PFOS	-0.000731466	0.009128920	-0.4590	5.7285
DVB-$BTMA^+$ / 1-octanol	PFOA	-0.014889897	-0.000314850	-9.3436	-0.1976
DVB-$BTMA^+$ / water	6:2 FTCA	-0.010842083	0.003032910	-6.8035	1.9032
DVB-$BTMA^+$ / water	PFHxA	-0.011272280	0.002673560	-7.0735	1.6777
DVB-$BTMA^+$ / water	PFOA	-0.015206944	-0.000723310	-9.5425	-0.4539

Model (solvent)	PFAS	ΔE (Eh)	ΔG (Eh)	ΔE (kcal/mol)	ΔG (kcal/mol)
DVB-BTMA$^+$ / water	PFOS	-0.014530749	0.001665280	-9.1182	1.0450

Table S3. BTMA$^+$ and extended monomer exchange energetics in water and 1-octanol. DVB-BTMA$^+$ denotes the extended styrene–divinylbenzene monomer. **(a)** Electronic source energies and **(b)** Gibbs source energies are in Eh. **(c)** Exchange electronic energies and free energies are in Eh and kcal/mol. The 1-octanol row reports PFOA. The same aqueous isolated-anion references are used in both models and both solvent cycles.

(a)

Species	Candidate	Electronic E (Eh)	G (Eh)	ΔE vs original	ΔG vs original	Exchange ΔE	Exchange ΔG
DVB-BTMA$^+$PFOA$^-$	Original	-2865.069247637	-2864.555186050	0.0000	0.0000	-9.3160	0.4103
DVB-BTMA$^+$PFOA$^-$	altA	-2865.069248657	-2864.555180010	-0.0006	0.0038	-9.3167	0.4141
DVB-BTMA$^+$PFOA$^-$	altB	-2865.069245795	-2864.554772700	0.0012	0.2594	-9.3149	0.6697
DVB-BTMA$^+$Cl$^-$	Original	-1372.331833816	-1371.893967240	0.0000	0.0000	-9.3160	0.4103
DVB-BTMA$^+$Cl$^-$	altA	-1372.331796155	-1371.894593530	0.0236	-0.3930	-9.3397	0.8033
DVB-BTMA$^+$Cl$^-$	altB	-1372.332017249	-1371.894344920	-0.1151	-0.2370	-9.2009	0.6473
PFOA$^-$	Original	-1953.067972856	-1953.023022170	0.0000	0.0000	-9.3160	0.4103
PFOA$^-$	altA	-1953.065660720	-1953.019642540	1.4509	2.1207	-10.7669	-1.7105
PFOA$^-$	altB	-1953.066809842	-1953.021861740	0.7298	0.7282	-10.0458	-0.3179

(b)

Species	Candidate	GOAT index	Search ΔE (kcal/mol)	Starting RMSD (Å)	Refined RMSD (Å)
DVB-BTMA$^+$PFOA$^-$	Original	0	0.0000	0.0000	0.0000
DVB-BTMA$^+$PFOA$^-$	altA	1	0.0211	0.1465	0.0069
DVB-BTMA$^+$PFOA$^-$	altB	3	0.2153	0.4051	0.0540
DVB-BTMA$^+$Cl$^-$	Original	0	0.0000	0.0000	0.0000
DVB-BTMA$^+$Cl$^-$	altA	2	0.0902	1.1987	1.2465
DVB-BTMA$^+$Cl$^-$	altB	3	0.2039	1.1365	1.2179
PFOA$^-$	Original	0	0.0000	0.0000	0.0000
PFOA$^-$	altA	1	0.1329	1.6125	1.6778
PFOA$^-$	altB	2	0.3760	1.7103	1.8885

Table S4. Aqueous r^{2}SCAN-3c conformer sensitivity of the extended-monomer PFOA exchange cycle. (a) Source energies and one-species substitutions, with all other species fixed at the original candidates. Electronic and Gibbs energies are in Eh. Differences and exchange values are in kcal/mol. **(b)** Zero-based GOAT ensemble indices, search-level relative energies, and symmetry-aware heavy-atom RMSDs relative to the original candidate. RMSD uses rigid alignment with element- and connectivity-preserving atom permutations. The search energy reference is the original GOAT minimum for that species. Quasi-RRHO used a 100 cm^{-1} reference frequency and a separate 1 cm^{-1} cutoff.

PFOA C–N (Å)	N–Cl (Å)	Above minimum (kcal/mol)	Absolute energy (Eh)
2.679	4.631	40.926	-133.57827399359
2.679	5.409	42.429	-133.57587985272
2.679	6.187	43.405	-133.57432375932
2.679	6.964	43.111	-133.57479152964
2.679	7.742	42.022	-133.57652733888
2.679	8.520	41.220	-133.57780619788
2.679	9.298	41.130	-133.57794898479
2.679	10.075	41.517	-133.57733182701
2.679	10.853	42.312	-133.57606531779
2.679	11.631	34.664	-133.58825399628
3.457	4.631	8.410	-133.63009132792
3.457	5.409	9.930	-133.62766936725
3.457	6.187	10.581	-133.62663244588
3.457	6.964	10.130	-133.62735045952
3.457	7.742	10.656	-133.62651224831
3.457	8.520	11.362	-133.62538750575
3.457	9.298	10.296	-133.62708620757
3.457	10.075	10.510	-133.62674472431
3.457	10.853	8.467	-133.63000130749
3.457	11.631	8.609	-133.62977453459
4.235	4.631	3.175	-133.63843496506
4.235	5.409	3.871	-133.63732494860
4.235	6.187	1.813	-133.64060462077
4.235	6.964	1.711	-133.64076672201
4.235	7.742	1.541	-133.64103810311
4.235	8.520	0.000	-133.64349397508
4.235	9.298	0.067	-133.64338797696
4.235	10.075	0.606	-133.64252813056
4.235	10.853	2.424	-133.63963098983
4.235	11.631	2.297	-133.63983344223
5.012	4.631	2.792	-133.63904488319
5.012	5.409	2.893	-133.63888388844
5.012	6.187	3.432	-133.63802494780
5.012	6.964	3.236	-133.63833751035
5.012	7.742	1.649	-133.64086576227
5.012	8.520	2.110	-133.64013180323
5.012	9.298	2.872	-133.63891725222
5.012	10.075	3.519	-133.63788588160
5.012	10.853	2.599	-133.63935161435
5.012	11.631	3.745	-133.63752547495
5.790	4.631	4.109	-133.63694621349

PFOA C–N (Å)	N–Cl (Å)	Above minimum (kcal/mol)	Absolute energy (Eh)
5.790	5.409	4.629	-133.63611789677
5.790	6.187	4.807	-133.63583343922
5.790	6.964	5.157	-133.63527534068
5.790	7.742	3.047	-133.63863881837
5.790	8.520	2.571	-133.63939634002
5.790	9.298	3.051	-133.63863145355
5.790	10.075	3.861	-133.63734177458
5.790	10.853	4.863	-133.63574424214
5.790	11.631	1.278	-133.64145805533
6.568	4.631	4.138	-133.63689986325
6.568	5.409	6.247	-133.63353914589
6.568	6.187	6.891	-133.63251290479
6.568	6.964	5.895	-133.63409898171
6.568	7.742	4.732	-133.63595372346
6.568	8.520	3.497	-133.63792068242
6.568	9.298	4.209	-133.63678580039
6.568	10.075	5.267	-133.63509989723
6.568	10.853	6.303	-133.63344954309
6.568	11.631	5.603	-133.63456480533
7.346	4.631	4.839	-133.63578303194
7.346	5.409	6.684	-133.63284247878
7.346	6.187	7.803	-133.63105974364
7.346	6.964	7.518	-133.63151256428
7.346	7.742	7.306	-133.63185110113
7.346	8.520	5.380	-133.63491980902
7.346	9.298	5.966	-133.63398655902
7.346	10.075	7.172	-133.63206433337
7.346	10.853	6.997	-133.63234425111
7.346	11.631	7.191	-133.63203499185
8.123	4.631	4.366	-133.63653633834
8.123	5.409	6.804	-133.63265157506
8.123	6.187	8.890	-133.62932734397
8.123	6.964	8.113	-133.63056431311
8.123	7.742	7.626	-133.63134123876
8.123	8.520	5.602	-133.63456685770
8.123	9.298	5.634	-133.63451551546
8.123	10.075	6.766	-133.63271208126
8.123	10.853	6.772	-133.63270239755
8.123	11.631	7.614	-133.63136031905
8.901	4.631	5.065	-133.63542172636
8.901	5.409	6.780	-133.63268982310

PFOA C–N (Å)	N–Cl (Å)	Above minimum (kcal/mol)	Absolute energy (Eh)
8.901	6.187	7.932	-133.63085314727
8.901	6.964	8.743	-133.62956069436
8.901	7.742	8.420	-133.63007613172
8.901	8.520	7.420	-133.63166878909
8.901	9.298	8.054	-133.63065857311
8.901	10.075	6.170	-133.63366073596
8.901	10.853	6.724	-133.63277888397
8.901	11.631	7.719	-133.63119263263
9.679	4.631	5.907	-133.63408073565
9.679	5.409	7.522	-133.63150697989
9.679	6.187	8.181	-133.63045606274
9.679	6.964	8.228	-133.63038110024
9.679	7.742	8.217	-133.63039993498
9.679	8.520	7.097	-133.63218424109
9.679	9.298	8.312	-133.63024829497
9.679	10.075	8.576	-133.62982733349
9.679	10.853	8.335	-133.63021202387
9.679	11.631	8.572	-133.62983302573

Table S5. Constrained PFOA carboxyl C–N and N–Cl two-distance potential energy surface. The 100 sampled geometries were optimized with xTB/ALPB(water) under the two listed distance constraints. Relative energies use the lowest sampled grid energy, −133.643493975080 Eh, as zero.

Category	Property	Value	Units or definition
Oligomer model	Name	pVBTMA12	Hydrogen-capped 12-unit poly(vinylbenzyltrimethylammonium) segment
Oligomer model	Formula	C144H218N12	
Oligomer model	Formal charge	12	elementary charge
Oligomer model	Atoms	374	atoms
Oligomer model	Heavy atoms	156	atoms
Oligomer model	Bonds	385	bonds
Oligomer model	Quaternary ammonium sites	12	sites
Oligomer model	Aromatic rings	12	rings
Oligomer model	Assigned backbone stereocenters	11	stereocenters
Charge model	AM1-BCC source oligomer	pVBTMA5	Capped five-unit model
Charge model	Repeat charge normalization	1	elementary charge per repeat
Charge model	Transferred chain charge	12	elementary charge
Force field	Organic atom types	GAFF2	
Force field	Full-chain parmchk2 flagged lines	0	lines
Force field	Dry topology connectivity	PASS	Source bonds reproduced
Chloride-form system	Water model	TIP3P	
Chloride-form system	Solvent buffer	15	Å
Chloride-form system	Water molecules	5183	molecules
Chloride-form system	Sodium ions	19	ions
Chloride-form system	Chloride ions	31	12 neutralizing ions plus 19 added-salt ions
Chloride-form system	Nominal added NaCl	0.1536	mol/L
PFOA-associated system	pVBTMA12 molecules	1	molecule
PFOA-associated system	PFOA molecules	1	deprotonated molecule; charge −1
PFOA-associated system	Sodium ions	19	ions
PFOA-associated system	Chloride ions	30	11 neutralizing ions plus 19 added-salt ions
PFOA-associated system	Water molecules	5163	molecules
PFOA-associated system	Total atoms	15937	atoms
PFOA-associated system	Net charge	0	elementary charge
PFOA-associated system	Initial selected repeat	9	repeat index
PFOA-associated system	Initial nearest O-to-N distance	4.4296	Å
PFOA-associated system	Waters removed for overlap	20	molecules

Table S6a. Twelve-unit pVBTMA oligomer and solvated PFOA-associated system definition. The finite, hydrogen-capped oligomer has twelve quaternary ammonium sites and a formal charge of 12+. The complete simulation box is electrically neutral with the specified ions.

Table S6b. Structural and parameterization checks for pVBTMA$_{12}$. Results refer to the defined oligomer and the chloride-form solvated precursor, as indicated.

Check	Criterion	Recorded result
Composition	Match the model definition	$C_{144}H_{218}N_{12}$, formal charge +12, 156 heavy atoms, one connected component, no dummy atoms. All three generated SDFs passed.
Chemical graph	Match constitution and site counts	All three SDFs matched the reference constitution, with 12 quaternary ammonium sites and 12 aromatic six-membered rings.
Stereochemistry	Preserve the specified stereoisomer	All three SDFs passed the exact-stereoisomer comparison. The model specifies 11 backbone stereocenters.
Initial geometry	Report nonbonded heavy-atom separation	Minimum distances: 2.075 Å after generation, 2.368 Å after UFF minimization, and 2.353 Å after optional MMFF94 minimization. UFF geometry was used for parameterization.
Atom mapping	Complete mapping and matching atom roles	All 374 mapping entries formed a bijection. Mapped elements and bond pairs agreed. Repeat-role multiplicity checks passed for all 12 repeats.
Charge transfer	Normalize each repeat to +1	The reduced-model serialized charge sum was +4.995001 e. Normalized head, internal, and tail libraries each sum to +1 e. Before serialization, all transferred repeat sums met 10^{-10} e tolerance and the chain summed to +12 e.
Serialized charge	Report residual and acceptance tolerance	Production MOL2: +11.999994 e. The strict 10^{-6} e test was not met. The residual, −0.000006 e, was within the documented serialization bound of 0.000187 e. Dry topology: +11.99999404 e, within its 10^{-5} e tolerance.
Charge comparison	RMSE ≤0.010 e and maximum difference ≤0.040 e for each group	Transferred versus direct whole-chain AM1-BCC charges passed for all groups. Overall RMSE: 0.004725 e. Largest group RMSE: 0.007295 e. Maximum absolute difference: 0.022813 e.
Production connectivity	Match source atom and bond indices	374 atoms, 385 bonds, 167 heavy-atom bonds, one component, and 12 four-coordinate nitrogen atoms. Indexed element and bond-pair comparisons passed.
Parameter coverage	No flagged missing GAFF2 terms	Full-chain parmchk2 assessment reported zero flagged lines in the generated parameter file.
Dry topology	Reproduce source graph and expected charge	374 atoms and 385 bonds. Topology and MOL2 bond-index sets matched. No periodic box was present, as expected for this check.
Chloride-form precursor	Match composition, ions, and periodic box	15,973 atoms, 5,183 waters, 19 Na^+ and 31 Cl^-. Charge residual: −0.00000596 e. Nominal added NaCl: 0.153586 M for a 0.150 M target. Polymer, ions, salt, box, water, and LEaP checks passed.

The SDF checks cover the generated, UFF-minimized, and optional MMFF94-minimized structures. Indexed MOL2 and topology comparisons check atom identities and bond pairs, not bond orders or stereochemistry. Minimum distances are limited clash diagnostics. Charge comparison tests the transfer approximation rather than independent AM1-BCC accuracy. The chloride-form precursor differs from the PFOA-associated production system in Table S6a. Sampling diagnostics are reported separately in Tables S8–S11.

Scope	Parameter	Value	Units or definition
Common	Software	NAMD3	AMBER topology and coordinate mode
Common	Ensemble	NPT	
Common	Temperature	310.1500	K
Common	Pressure	1.0132	bar
Common	Time step	2	fs
Common	Electrostatics	Particle mesh Ewald	Grid spacing 1 Å
Common	Nonbonded cutoff	9	Å
Common	Pairlist distance	11	Å
Common	Switching	Off	
Common	Lennard-Jones long-range correction	On	
Common	Hydrogen-bond constraint	rigidBonds all	
Common	Langevin damping	1	ps−1
Common	Piston period	100	fs
Common	Piston decay	50	fs
Common	Restraints	None	
Common	Equilibration per replica	0.5000	ns
Common	Production per replica	10	ns
Common	Production segments	10	1 ns restartable segments per replica
Common	Saved-frame interval	2	ps
Common	Production frames per replica	5000	frames
Replica 1	Production duration	10	ns
Replica 1	Production frames	5000	frames
Replica 2	Production duration	10	ns
Replica 2	Production frames	5000	frames
Replica 3	Production duration	10	ns
Replica 3	Production frames	5000	frames
Analysis	Block duration	1	ns
Analysis	Blocks per replica	10	blocks
Analysis	ESS screen	20	minimum effective sample size
Analysis	Early-to-late shift screen	0.5000	maximum within-replica SD
Analysis	Replica-spread screen	1	maximum pooled within-replica SD

Table S7. Molecular dynamics protocol and three production replicas. Each 10 ns production replica followed separate equilibration. Analysis sampled 5,000 frames per replica at 2 ps intervals.

Replica	Metric	n	Mean	SD	Min	Max	IAT (frames)	IAT (ps)	ESS
replica_01	Oligomer RMSD	5000	4.685	0.815	0.000	7.041	172.092	344.184	14.5
replica_01	Oligomer Rg	5000	9.335	0.268	8.331	10.082	139.838	279.677	17.9
replica_01	End-to-end	5000	24.714	2.353	15.830	29.869	186.311	372.621	13.4
replica_01	Head–selected N	5000	8.721	2.912	3.526	19.210	164.758	329.516	15.2
replica_01	Head–any N	5000	5.165	0.916	3.268	9.653	11.775	23.550	212.3
replica_01	PFOA–oligomer	5000	3.006	0.144	2.584	3.748	45.739	91.477	54.7
replica_01	Oligomer image	5000	29.271	2.497	19.198	34.865	112.478	224.955	22.2
replica_01	PFOA image	5000	45.625	1.439	40.742	48.005	71.555	143.110	34.9
replica_01	Tail contacts	5000	16.271	4.646	1.000	31.000	149.502	299.005	16.7
replica_01	Tail waters	5000	37.405	5.747	21.000	63.000	142.822	285.644	17.5
replica_02	Oligomer RMSD	5000	3.804	1.009	0.000	6.464	475.396	950.792	5.3
replica_02	Oligomer Rg	5000	9.312	0.264	8.444	10.120	217.095	434.190	11.5
replica_02	End-to-end	5000	23.716	1.597	17.767	27.960	71.358	142.716	35.0
replica_02	Head–selected N	5000	11.158	4.630	3.530	23.335	687.945	1375.890	3.6
replica_02	Head–any N	5000	5.148	0.876	3.314	8.231	15.277	30.553	163.6
replica_02	PFOA–oligomer	5000	3.014	0.140	2.611	3.719	8.676	17.353	288.1
replica_02	Oligomer image	5000	28.026	3.098	18.323	35.895	123.879	247.759	20.2
replica_02	PFOA image	5000	45.477	1.553	40.584	48.109	99.806	199.613	25.0
replica_02	Tail contacts	5000	15.492	4.124	1.000	32.000	60.602	121.203	41.3
replica_02	Tail waters	5000	36.057	4.967	22.000	53.000	92.829	185.657	26.9
replica_03	Oligomer RMSD	5000	4.049	0.832	0.000	6.263	262.098	524.196	9.5
replica_03	Oligomer Rg	5000	9.293	0.216	8.620	10.014	124.838	249.677	20.0
replica_03	End-to-end	5000	23.231	2.772	15.147	28.072	422.469	844.939	5.9
replica_03	Head–selected N	5000	8.009	2.510	3.472	18.954	375.850	751.701	6.7
replica_03	Head–any N	5000	5.055	0.910	3.298	8.181	65.172	130.344	38.4
replica_03	PFOA–oligomer	5000	2.974	0.123	2.582	3.508	1.462	2.924	1710.2
replica_03	Oligomer image	5000	30.241	2.684	21.203	35.983	85.642	171.284	29.2
replica_03	PFOA image	5000	45.104	1.787	40.868	48.008	134.068	268.136	18.6
replica_03	Tail contacts	5000	18.008	4.281	3.000	35.000	124.667	249.334	20.1
replica_03	Tail waters	5000	36.226	4.894	21.000	51.000	147.703	295.407	16.9

Table S8. Per-replica molecular dynamics summaries. Each row summarizes 5,000 sampled frames. IAT is integrated autocorrelation time. ESS is effective sample size. RMSD, radius of gyration (R_g), end-to-end, head–N, PFOA–oligomer, and periodic self-image metrics are distances in Å. Head–selected N uses the initially selected ammonium site. Head–any N uses the nearest of all twelve sites. Tail contacts and waters are counts. IAT includes half the zero-lag contribution and the initial positive normalized autocorrelation sequence. ESS is n divided by twice IAT in frames.

Replica	1 ns block	Metric	Mean	SD
replica_01	1	Oligomer RMSD	3.037	1.105
replica_01	2	Oligomer RMSD	5.083	0.670
replica_01	3	Oligomer RMSD	4.865	0.441
replica_01	4	Oligomer RMSD	4.246	0.463
replica_01	5	Oligomer RMSD	4.602	0.297
replica_01	6	Oligomer RMSD	5.367	0.481
replica_01	7	Oligomer RMSD	4.975	0.285
replica_01	8	Oligomer RMSD	4.882	0.404
replica_01	9	Oligomer RMSD	4.750	0.293
replica_01	10	Oligomer RMSD	5.043	0.297
replica_01	1	Oligomer Rg	9.581	0.177
replica_01	2	Oligomer Rg	9.350	0.239
replica_01	3	Oligomer Rg	9.464	0.178
replica_01	4	Oligomer Rg	9.398	0.278
replica_01	5	Oligomer Rg	9.308	0.210
replica_01	6	Oligomer Rg	8.901	0.250
replica_01	7	Oligomer Rg	9.338	0.215
replica_01	8	Oligomer Rg	9.267	0.156
replica_01	9	Oligomer Rg	9.323	0.179
replica_01	10	Oligomer Rg	9.419	0.168
replica_01	1	End-to-end	23.350	2.100
replica_01	2	End-to-end	23.665	1.795
replica_01	3	End-to-end	26.507	1.295
replica_01	4	End-to-end	26.460	2.003
replica_01	5	End-to-end	24.721	1.644
replica_01	6	End-to-end	20.880	2.521
replica_01	7	End-to-end	24.947	1.720
replica_01	8	End-to-end	24.938	1.106
replica_01	9	End-to-end	25.724	1.100
replica_01	10	End-to-end	25.949	1.025
replica_01	1	Head–selected N	8.141	1.410
replica_01	2	Head–selected N	6.931	1.570
replica_01	3	Head–selected N	7.680	1.484
replica_01	4	Head–selected N	8.457	2.341
replica_01	5	Head–selected N	9.280	2.455
replica_01	6	Head–selected N	8.903	1.211
replica_01	7	Head–selected N	8.548	2.039
replica_01	8	Head–selected N	6.334	1.304
replica_01	9	Head–selected N	9.684	4.133
replica_01	10	Head–selected N	13.257	3.167
replica_01	1	Head–any N	5.176	0.814

Replica	1 ns block	Metric	Mean	SD
replica_01	2	Head–any N	5.160	0.882
replica_01	3	Head–any N	5.258	0.973
replica_01	4	Head–any N	5.350	0.870
replica_01	5	Head–any N	5.443	0.807
replica_01	6	Head–any N	5.302	0.924
replica_01	7	Head–any N	5.080	0.989
replica_01	8	Head–any N	4.988	0.789
replica_01	9	Head–any N	4.905	0.936
replica_01	10	Head–any N	4.986	1.003
replica_01	1	PFOA–oligomer	2.975	0.124
replica_01	2	PFOA–oligomer	2.946	0.115
replica_01	3	PFOA–oligomer	2.962	0.124
replica_01	4	PFOA–oligomer	2.984	0.124
replica_01	5	PFOA–oligomer	2.985	0.126
replica_01	6	PFOA–oligomer	2.991	0.133
replica_01	7	PFOA–oligomer	3.068	0.152
replica_01	8	PFOA–oligomer	3.000	0.127
replica_01	9	PFOA–oligomer	3.059	0.157
replica_01	10	PFOA–oligomer	3.087	0.170
replica_01	1	Oligomer image	29.056	1.973
replica_01	2	Oligomer image	30.814	1.556
replica_01	3	Oligomer image	30.204	1.543
replica_01	4	Oligomer image	29.502	2.106
replica_01	5	Oligomer image	28.110	3.358
replica_01	6	Oligomer image	27.739	1.762
replica_01	7	Oligomer image	27.366	2.758
replica_01	8	Oligomer image	29.503	2.047
replica_01	9	Oligomer image	31.456	1.328
replica_01	10	Oligomer image	28.957	2.396
replica_01	1	PFOA image	44.566	1.697
replica_01	2	PFOA image	46.014	0.509
replica_01	3	PFOA image	45.899	1.113
replica_01	4	PFOA image	46.422	1.164
replica_01	5	PFOA image	46.571	0.782
replica_01	6	PFOA image	45.641	1.117
replica_01	7	PFOA image	45.149	1.129
replica_01	8	PFOA image	46.286	0.725
replica_01	9	PFOA image	45.027	1.697
replica_01	10	PFOA image	44.670	1.911
replica_01	1	Tail contacts	16.336	2.961
replica_01	2	Tail contacts	18.042	3.081

Replica	1 ns block	Metric	Mean	SD
replica_01	3	Tail contacts	17.878	2.991
replica_01	4	Tail contacts	18.116	3.175
replica_01	5	Tail contacts	18.502	3.686
replica_01	6	Tail contacts	17.342	3.268
replica_01	7	Tail contacts	13.206	3.107
replica_01	8	Tail contacts	19.942	3.769
replica_01	9	Tail contacts	13.488	5.530
replica_01	10	Tail contacts	9.860	3.627
replica_01	1	Tail waters	38.924	3.959
replica_01	2	Tail waters	39.256	3.089
replica_01	3	Tail waters	39.366	3.481
replica_01	4	Tail waters	34.652	4.722
replica_01	5	Tail waters	34.612	4.832
replica_01	6	Tail waters	35.762	4.689
replica_01	7	Tail waters	37.868	3.792
replica_01	8	Tail waters	31.172	2.952
replica_01	9	Tail waters	38.580	7.520
replica_01	10	Tail waters	43.862	6.021
replica_02	1	Oligomer RMSD	2.326	0.551
replica_02	2	Oligomer RMSD	2.519	0.390
replica_02	3	Oligomer RMSD	3.641	0.965
replica_02	4	Oligomer RMSD	4.522	0.784
replica_02	5	Oligomer RMSD	5.107	0.441
replica_02	6	Oligomer RMSD	4.759	0.410
replica_02	7	Oligomer RMSD	4.095	0.371
replica_02	8	Oligomer RMSD	3.508	0.437
replica_02	9	Oligomer RMSD	3.664	0.342
replica_02	10	Oligomer RMSD	3.903	0.426
replica_02	1	Oligomer Rg	9.608	0.160
replica_02	2	Oligomer Rg	9.585	0.184
replica_02	3	Oligomer Rg	9.299	0.309
replica_02	4	Oligomer Rg	9.156	0.195
replica_02	5	Oligomer Rg	9.190	0.185
replica_02	6	Oligomer Rg	9.072	0.175
replica_02	7	Oligomer Rg	9.320	0.169
replica_02	8	Oligomer Rg	9.400	0.154
replica_02	9	Oligomer Rg	9.366	0.145
replica_02	10	Oligomer Rg	9.120	0.247
replica_02	1	End-to-end	23.899	1.230
replica_02	2	End-to-end	24.656	1.493
replica_02	3	End-to-end	24.024	1.669

Replica	1 ns block	Metric	Mean	SD
replica_02	4	End-to-end	23.992	1.201
replica_02	5	End-to-end	23.575	1.109
replica_02	6	End-to-end	21.957	1.482
replica_02	7	End-to-end	23.755	1.399
replica_02	8	End-to-end	23.938	1.433
replica_02	9	End-to-end	24.363	1.064
replica_02	10	End-to-end	22.998	1.949
replica_02	1	Head–selected N	10.335	2.168
replica_02	2	Head–selected N	7.766	2.204
replica_02	3	Head–selected N	7.815	2.581
replica_02	4	Head–selected N	6.331	1.730
replica_02	5	Head–selected N	7.395	2.231
replica_02	6	Head–selected N	9.611	3.365
replica_02	7	Head–selected N	13.372	2.070
replica_02	8	Head–selected N	16.303	1.870
replica_02	9	Head–selected N	18.950	1.972
replica_02	10	Head–selected N	13.699	2.334
replica_02	1	Head–any N	5.477	0.932
replica_02	2	Head–any N	5.350	0.851
replica_02	3	Head–any N	4.968	0.799
replica_02	4	Head–any N	5.112	0.846
replica_02	5	Head–any N	4.914	0.771
replica_02	6	Head–any N	5.287	0.986
replica_02	7	Head–any N	5.215	0.852
replica_02	8	Head–any N	5.452	0.934
replica_02	9	Head–any N	4.834	0.719
replica_02	10	Head–any N	4.875	0.731
replica_02	1	PFOA–oligomer	2.979	0.131
replica_02	2	PFOA–oligomer	2.964	0.113
replica_02	3	PFOA–oligomer	3.021	0.144
replica_02	4	PFOA–oligomer	2.988	0.129
replica_02	5	PFOA–oligomer	2.997	0.130
replica_02	6	PFOA–oligomer	3.047	0.146
replica_02	7	PFOA–oligomer	3.058	0.154
replica_02	8	PFOA–oligomer	3.025	0.140
replica_02	9	PFOA–oligomer	3.018	0.135
replica_02	10	PFOA–oligomer	3.038	0.147
replica_02	1	Oligomer image	26.986	2.270
replica_02	2	Oligomer image	25.762	3.193
replica_02	3	Oligomer image	25.873	3.145
replica_02	4	Oligomer image	28.707	2.082

Replica	1 ns block	Metric	Mean	SD
replica_02	5	Oligomer image	27.948	1.834
replica_02	6	Oligomer image	30.505	1.794
replica_02	7	Oligomer image	28.812	3.045
replica_02	8	Oligomer image	27.262	3.336
replica_02	9	Oligomer image	27.871	3.083
replica_02	10	Oligomer image	30.530	2.253
replica_02	1	PFOA image	44.320	1.655
replica_02	2	PFOA image	45.935	1.245
replica_02	3	PFOA image	45.443	1.332
replica_02	4	PFOA image	46.147	0.777
replica_02	5	PFOA image	44.014	1.460
replica_02	6	PFOA image	44.914	1.641
replica_02	7	PFOA image	46.291	1.022
replica_02	8	PFOA image	45.365	1.475
replica_02	9	PFOA image	46.619	1.165
replica_02	10	PFOA image	45.719	1.253
replica_02	1	Tail contacts	14.976	2.777
replica_02	2	Tail contacts	16.200	2.821
replica_02	3	Tail contacts	17.000	5.227
replica_02	4	Tail contacts	18.020	4.651
replica_02	5	Tail contacts	17.714	4.610
replica_02	6	Tail contacts	13.644	3.556
replica_02	7	Tail contacts	13.942	3.719
replica_02	8	Tail contacts	14.136	3.339
replica_02	9	Tail contacts	14.186	3.252
replica_02	10	Tail contacts	15.102	3.512
replica_02	1	Tail waters	39.880	3.400
replica_02	2	Tail waters	39.620	3.670
replica_02	3	Tail waters	33.264	5.484
replica_02	4	Tail waters	34.164	5.910
replica_02	5	Tail waters	32.090	3.932
replica_02	6	Tail waters	37.698	4.474
replica_02	7	Tail waters	37.360	3.878
replica_02	8	Tail waters	37.030	3.025
replica_02	9	Tail waters	36.960	3.557
replica_02	10	Tail waters	32.506	3.382
replica_03	1	Oligomer RMSD	4.540	1.112
replica_03	2	Oligomer RMSD	5.153	0.348
replica_03	3	Oligomer RMSD	4.367	0.490
replica_03	4	Oligomer RMSD	3.971	0.414
replica_03	5	Oligomer RMSD	3.352	0.308

Replica	1 ns block	Metric	Mean	SD
replica_03	6	Oligomer RMSD	4.326	0.364
replica_03	7	Oligomer RMSD	3.938	0.298
replica_03	8	Oligomer RMSD	2.982	0.852
replica_03	9	Oligomer RMSD	3.487	0.586
replica_03	10	Oligomer RMSD	4.375	0.265
replica_03	1	Oligomer Rg	9.461	0.214
replica_03	2	Oligomer Rg	9.306	0.159
replica_03	3	Oligomer Rg	9.255	0.154
replica_03	4	Oligomer Rg	9.314	0.182
replica_03	5	Oligomer Rg	9.224	0.184
replica_03	6	Oligomer Rg	9.367	0.141
replica_03	7	Oligomer Rg	9.410	0.123
replica_03	8	Oligomer Rg	9.355	0.215
replica_03	9	Oligomer Rg	9.249	0.227
replica_03	10	Oligomer Rg	8.984	0.128
replica_03	1	End-to-end	23.900	1.381
replica_03	2	End-to-end	23.429	1.260
replica_03	3	End-to-end	24.193	1.197
replica_03	4	End-to-end	25.457	1.061
replica_03	5	End-to-end	25.065	0.838
replica_03	6	End-to-end	24.020	1.216
replica_03	7	End-to-end	25.281	1.068
replica_03	8	End-to-end	23.808	2.303
replica_03	9	End-to-end	18.795	1.406
replica_03	10	End-to-end	18.358	1.366
replica_03	1	Head–selected N	12.972	1.757
replica_03	2	Head–selected N	9.983	2.231
replica_03	3	Head–selected N	8.563	1.060
replica_03	4	Head–selected N	7.196	1.435
replica_03	5	Head–selected N	7.122	1.374
replica_03	6	Head–selected N	7.126	1.351
replica_03	7	Head–selected N	5.724	1.405
replica_03	8	Head–selected N	7.820	1.338
replica_03	9	Head–selected N	7.809	1.108
replica_03	10	Head–selected N	5.774	1.304
replica_03	1	Head–any N	5.195	0.978
replica_03	2	Head–any N	5.488	1.062
replica_03	3	Head–any N	5.675	0.933
replica_03	4	Head–any N	5.110	0.900
replica_03	5	Head–any N	4.712	0.739
replica_03	6	Head–any N	5.244	0.880

Replica	1 ns block	Metric	Mean	SD
replica_03	7	Head–any N	4.654	0.576
replica_03	8	Head–any N	4.881	0.694
replica_03	9	Head–any N	4.921	0.897
replica_03	10	Head–any N	4.665	0.692
replica_03	1	PFOA–oligomer	3.010	0.139
replica_03	2	PFOA–oligomer	2.982	0.123
replica_03	3	PFOA–oligomer	2.975	0.119
replica_03	4	PFOA–oligomer	2.962	0.121
replica_03	5	PFOA–oligomer	2.967	0.122
replica_03	6	PFOA–oligomer	2.969	0.127
replica_03	7	PFOA–oligomer	2.965	0.120
replica_03	8	PFOA–oligomer	2.991	0.117
replica_03	9	PFOA–oligomer	2.965	0.119
replica_03	10	PFOA–oligomer	2.949	0.109
replica_03	1	Oligomer image	31.574	1.927
replica_03	2	Oligomer image	27.057	2.771
replica_03	3	Oligomer image	28.654	1.886
replica_03	4	Oligomer image	30.782	2.449
replica_03	5	Oligomer image	31.656	1.544
replica_03	6	Oligomer image	29.281	2.210
replica_03	7	Oligomer image	30.129	2.537
replica_03	8	Oligomer image	30.552	2.957
replica_03	9	Oligomer image	30.736	1.895
replica_03	10	Oligomer image	31.984	2.029
replica_03	1	PFOA image	43.822	1.325
replica_03	2	PFOA image	43.405	1.711
replica_03	3	PFOA image	45.109	1.278
replica_03	4	PFOA image	46.006	1.087
replica_03	5	PFOA image	44.118	1.463
replica_03	6	PFOA image	46.621	0.925
replica_03	7	PFOA image	42.992	1.464
replica_03	8	PFOA image	46.279	0.690
replica_03	9	PFOA image	46.296	0.756
replica_03	10	PFOA image	46.395	0.854
replica_03	1	Tail contacts	15.038	3.326
replica_03	2	Tail contacts	15.742	3.028
replica_03	3	Tail contacts	16.492	2.733
replica_03	4	Tail contacts	20.402	4.296
replica_03	5	Tail contacts	18.610	3.637
replica_03	6	Tail contacts	16.270	2.489
replica_03	7	Tail contacts	17.026	3.431

Replica	1 ns block	Metric	Mean	SD
replica_03	8	Tail contacts	18.154	2.893
replica_03	9	Tail contacts	17.858	3.103
replica_03	10	Tail contacts	24.488	4.449
replica_03	1	Tail waters	37.882	5.173
replica_03	2	Tail waters	39.316	2.918
replica_03	3	Tail waters	38.880	2.974
replica_03	4	Tail waters	34.126	4.519
replica_03	5	Tail waters	35.126	3.708
replica_03	6	Tail waters	39.972	3.167
replica_03	7	Tail waters	37.834	3.238
replica_03	8	Tail waters	33.464	2.857
replica_03	9	Tail waters	36.520	4.890
replica_03	10	Tail waters	29.140	3.058

Table S9. One-nanosecond block summaries from three 10 ns production replicas. Mean and SD are computed from sampled frames within each block. The ten metrics and their units follow Table S8. Blocks are indexed 1–10 separately for each replica.

Replica	Metric	ESS	Early mean	Late mean	Shift/SD	Replica range	Pooled SD	Spread ratio
replica_01	Oligomer Rg	17.9	9.581	9.419	0.607	0.042	0.250	0.169
replica_02	Oligomer Rg	11.5	9.608	9.120	1.845	0.042	0.250	0.169
replica_03	Oligomer Rg	20.0	9.461	8.984	2.212	0.042	0.250	0.169
replica_01	End-to-end	13.4	23.350	25.949	1.105	1.483	2.292	0.647
replica_02	End-to-end	35.0	23.899	22.998	0.564	1.483	2.292	0.647
replica_03	End-to-end	5.9	23.900	18.358	2.000	1.483	2.292	0.647
replica_01	Head–any N	212.3	5.176	4.986	0.208	0.110	0.901	0.122
replica_02	Head–any N	163.6	5.477	4.875	0.687	0.110	0.901	0.122
replica_03	Head–any N	38.4	5.195	4.665	0.582	0.110	0.901	0.122
replica_01	PFOA–oligomer	54.7	2.975	3.087	0.779	0.040	0.136	0.294
replica_02	PFOA–oligomer	288.1	2.979	3.038	0.421	0.040	0.136	0.294
replica_03	PFOA–oligomer	1710.2	3.010	2.949	0.495	0.040	0.136	0.294

Table S10. Numerical sampling diagnostics for selected molecular dynamics metrics. Early and late means and the standardized shift summarize within-replica behavior. Replica mean range, pooled within-replica SD, and their ratio summarize between-replica spread.

Replica	1 ns block	Fraction below 5 Å
replica_01	1	0.514
replica_01	2	0.538
replica_01	3	0.440
replica_01	4	0.410
replica_01	5	0.322
replica_01	6	0.462
replica_01	7	0.550
replica_01	8	0.552
replica_01	9	0.618
replica_01	10	0.568
replica_02	1	0.370
replica_02	2	0.376
replica_02	3	0.650
replica_02	4	0.566
replica_02	5	0.632
replica_02	6	0.428
replica_02	7	0.496
replica_02	8	0.364
replica_02	9	0.654
replica_02	10	0.628
replica_03	1	0.504
replica_03	2	0.388
replica_03	3	0.286
replica_03	4	0.566
replica_03	5	0.778
replica_03	6	0.418
replica_03	7	0.766
replica_03	8	0.684
replica_03	9	0.626
replica_03	10	0.778

Table S11. PFOA headgroup contact fraction near ammonium by one-nanosecond block. The fraction is the proportion of sampled frames with a PFOA carboxylate oxygen at a distance of less than 5 Å from any oligomer ammonium nitrogen.
